# Determinants of renal cell carcinoma invasion and metastatic competence

**DOI:** 10.1038/s41467-021-25918-4

**Published:** 2021-10-04

**Authors:** Kangsan Kim, Qinbo Zhou, Alana Christie, Christina Stevens, Yuanqing Ma, Oreoluwa Onabolu, Suneetha Chintalapati, Tiffani Mckenzie, Vanina Toffessi Tcheuyap, Layton Woolford, He Zhang, Nirmish Singla, Pravat Kumar Parida, Mauricio Marquez-Palencia, Ivan Pedrosa, Vitaly Margulis, Arthur Sagalowsky, Zhiqun Xie, Tao Wang, Steffen Durinck, Zora Modrusan, Somasekar Seshagiri, Payal Kapur, James Brugarolas, Srinivas Malladi

**Affiliations:** 1 Department of Pathology, University of Texas Southwestern Medical Center, Dallas, TX 75390 USA; 2 Harold C. Simmons Comprehensive Cancer Center, University of Texas Southwestern Medical Center, Dallas, TX 75390 USA; 3 Quantitative Biomedical Research Center, Department of Population and Data Sciences, University of Texas Southwestern Medical Center, Dallas, TX 75390 USA; 4 Kidney Cancer Program, Simmons Comprehensive Cancer Center, The University of Texas Southwestern Medical Center, Dallas, TX 75390 USA; 5 Hematology-Oncology Division, Department of Internal Medicine, University of Texas Southwestern Medical Center, Dallas, TX 75390 USA; 6 Bioinformatics Core Facility, The University of Texas Southwestern Medical Center, Dallas, TX 75390 USA; 7 Department of Urology, The University of Texas Southwestern Medical Center, Dallas, TX 75390 USA; 8 Department of Radiology, University of Texas Southwestern Medical Center, Dallas, TX 75390 USA; 9 Center for the Genetics of Host Defense, University of Texas Southwestern Medical Center, Dallas, TX 75390 USA; 10 Molecular Biology Department, Genentech, Inc., South San Francisco, CA 94080 USA

**Keywords:** Cancer genomics, Metastasis, Renal cell carcinoma

## Abstract

Metastasis is the principal cause of cancer related deaths. Tumor invasion is essential for metastatic spread. However, determinants of invasion are poorly understood. We addressed this knowledge gap by leveraging a unique attribute of kidney cancer. Renal tumors invade into large vessels forming tumor thrombi (TT) that migrate extending sometimes into the heart. Over a decade, we prospectively enrolled 83 ethnically-diverse patients undergoing surgical resection for grossly invasive tumors at UT Southwestern Kidney Cancer Program. In this study, we perform comprehensive histological analyses, integrate multi-region genomic studies, generate in vivo models, and execute functional studies to define tumor invasion and metastatic competence. We find that invasion is not always associated with the most aggressive clone. Driven by immediate early genes, invasion appears to be an opportunistic trait attained by subclones with diverse oncogenomic status in geospatial proximity to vasculature. We show that not all invasive tumors metastasize and identify determinants of metastatic competency. TT associated with metastases are characterized by higher grade, mTOR activation and a particular immune contexture. Moreover, TT grade is a better predictor of metastasis than overall tumor grade, which may have implications for clinical practice.

## Abstract (web summary)

Tumour thrombi (TT) are intravascular extensions of renal cell carcinomas (RCC) which often lead to distant metastases. Here the authors examine the determinants of vascular invasion and metastasis in a unique cohort of RCC patients with TT using multi-region genomics and in vivo models.

## Introduction

Metastasis is the major cause of cancer-related deaths. It is a complex process involving tumor cell invasion, dissemination through the vasculature or lymphatic vessels, extravasation, and colonization of distant organs^1–3^. While invasion is thought to be a rate limiting step, its relative contribution to the metastatic process is poorly understood. An important limitation in delineating determinants of metastatic competence associated with invasion is a lack of suitable physiologically relevant experimental systems and the inability to track metastatic progression in real time. In vitro systems hardly recapitulate the physiological complexity of the metastatic process, and in vivo studies are limited by the often microscopic nature of intravascular capillary tumor extensions. Constraints imposed by necessarily reductionist experimental approaches and laboratory models have impeded progress in delineating determinants of invasion and metastatic competence^2,4,5^.

The epithelial to mesenchymal transition (EMT) developmental program is known to be hijacked by cancer cells to invade and initiate metastases^2,6–8^. Cancer cell invasion is not restricted to single cells and collective cell migration may be observed in histopathological samples; however, its contribution to metastatic progression is poorly understood^3,9,10^. It is believed that invasive cells exist in several distinct cellular states described as hybrid or partial EMT depending on the activation status of EMT transcription factors^11–13^.

Renal cancer offers a unique experimental paradigm to dissect the link between vascular invasion and metastases. Renal cell carcinoma (RCC), the most common kidney tumor, grossly invades into large vessels. Tumor extensions (so called tumor thrombi [TT]) into the renal vein are observed in approximately 15% of RCC patients. These TT are able to traverse extensive distances arriving sometimes to the heart. This directional process offers a unique, but yet untapped opportunity to understand the relationship between invasion, migration and metastases.

Importantly, the extent of TT invasion drives tumor staging and prognosis. Level I thrombi invade into the renal vein and its branches (stage pT3a); level II TT extend into the inferior vena cava (IVC) up to the hepatic veins (pT3b); level III TT approximate the diaphragm (also pT3b); and level IV TT extend into the heart (pT3c). The level of TT has been shown to have prognostic significance with 5-year survival rates of 43%, 37% and 22% for pT3a, pT3b and pT3c, respectively^14^.

RCC with TT are typically managed surgically with removal of the kidney tumor and TT. This sometimes requires extracorporeal circulation with cardiac bypass to remove the TT from the heart. Remarkably, despite gross invasion into the largest vein in the body (the IVC), and extensive migration sometimes into the heart, not all RCC TT lead to metastasis^15^.

Here, we report the results of extensive analyses of a prospectively enrolled cohort of 83 ethnically-diverse patients with a renal mass and associated TT scheduled to undergo surgical resection at UT Southwestern Medical Center Kidney Cancer Program over a 10-year period. We performed extensive histological and integrated genomic analyses, implanted TT in mice and  executed functional studies. The study is divided into two parts. Initially we focus on evaluating determinants of invasion by comparing TT and the corresponding primary tumors (PT). Subsequently, we explore determinants of metastatic competence by comparing TT from patients that developed metastases to the ones that did not.

## Results

### Spatially resolved multi-region sampling and co-registered integrated genomic analyses of RCC with intravascular tumor extension

**Fig. 1:**
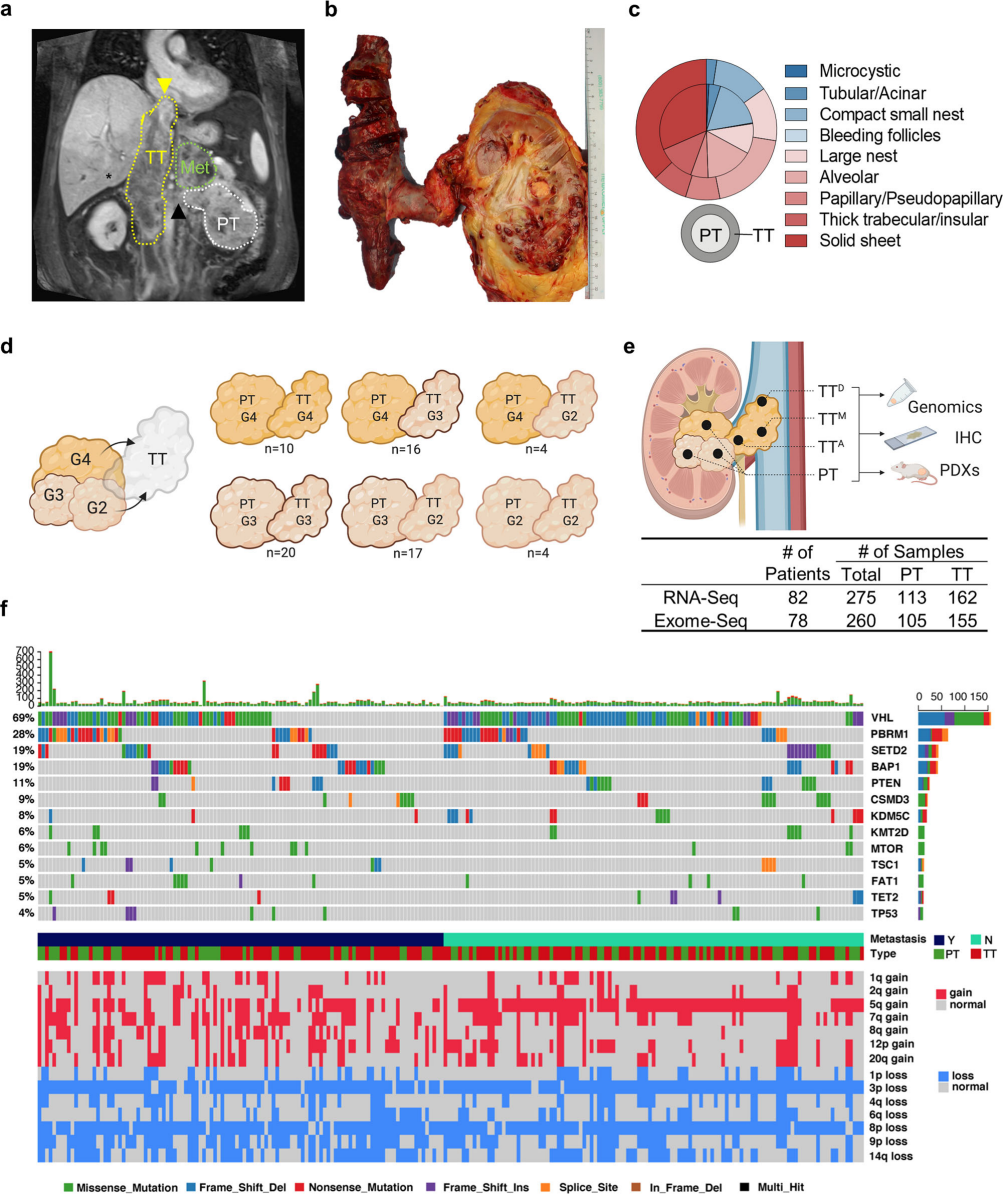
Characterization of invasive intravascular RCC tumors. **a** MRI scan of a patient with a clear cell renal cell carcinoma (ccRCC) and inferior vena cava (IVC) thrombus. Coronal T1-weighted fat saturated contrast-enhanced spoiled gradient echo image acquired during the nephrographic phase shows a large left renal mass (white dotted line) extending into the proximal renal vein (black arrowhead). Tumor thrombus extends into the IVC (yellow dotted line) with its distal tip (yellow arrowhead) above the diaphragm and entering the right atrium (Level IV). A large heterogeneous left adrenal metastasis (green dotted line) is present. **b** Macroscopic image of a primary tumor (PT) and tumor thrombus (TT) from the same patient. **c** Summarized architectural patterns (indolent (microcystic) to aggressive (solid sheet)) within PT (inner circle) and TT (outer circle) in 71 ccRCC patients with intravascular TT. **d** Representation of microscopic analysis of PT-TT paired samples based on grade. **e** Illustration showing multi-region sampling of invasive tumor samples for sequencing, PDX generation, and histological analyses. PT primary tumor, TT-A adjacent thrombus, TT-M middle thrombus, TT-D distal thrombus. **f** Overview of mutations, CNVs, and clinical parameters for ccRCC patients.

We prospectively enrolled 83 patients with a renal mass and TT scheduled to undergo surgical resection. Supplementary Table 1 summarizes the clinicopathological features of this cohort. All but one tumor (a leiomyosarcoma) were RCCs (73 Clear Cell, 1 Papillary, 1 Chromophobe, and 7 Unclassified) (Fig. 1a, b). We performed extensive histological analyses of RCC PT and TT. RCCs are notoriously heterogeneous^16,17^, and exhibited multiple architectural patterns (Fig. 1c and Supplementary Fig. 1a, b).

Comprehensive microscopic examination across PT and TT from 71 patients revealed that PTs were more heterogenous than TT. Fifty percent of PTs (36/71) showed 3-4 different architectural patterns whereas over 90% of TT (65/71) had 1–2 patterns (Supplementary Fig. 1c). While both indolent and aggressive patterns were observed in PT, aggressive architectural patternspredominated in TT (Fig. 1c and Supplementary Fig. 1c; Supplementary Table. 2).

We then focused on tumor grade, which is a critical determinant of prognosis^18^. Tumor grade is largely based on nucleolar prominence and scored from low (grade 1 and 2) to high (grade 3 and 4). As for architectural patterns, tumor grade varies from area to area in tumors. In routine clinical practice, overall tumor grade is based on the highest-grade area observed. Comparative analyses of tumor grade between PT and TT revealed discrepancies in tumor grade (Supplementary Fig. 1d). Unexpectedly, TT were not always colonized by the highest grade clone in the PT. In fact, among PT of grade 3 and 4, the TT was of lower grade in 45% and 66% of cases, respectively (Fig. 1d). These data show that TT are not always seeded by the most aggressive clone.

We collected 277 spatially resolved paired PT and TT samples. Samples were selected from macroscopically diverse areas (Fig. 1a, b). We leveraged the directionality afforded by TT. A sample was often collected adjacent to the primary tumor (TT^A^), at the distal leading tip (TT^D^) and somewhere in the middle (TT^M^) (Fig. 1e). Several samples from the PT were also gathered. When accessible, matched metastatic lesions (M; Met) were similarly collected (*n* = 6 patients).

Tumor samples were used for integrated genomic analyses and transplanted into mice to generate live tumor models (patient-derived xenograft [PDX] or tumorgraft [TG] models; Fig. 1e). To accurately integrate genomic and transcriptomic findings and enable histological co-registration, simultaneous isolation of DNA and RNA was performed from individual samples characterized histologically through immediately flanking sections^19^. Whole-exome sequencing (WES) analyses were performed on 260 matched PT/TT from 78 patients and RNA-seq from 275 matched PT/TT from 82 patients (Fig. 1e).

### Oncogenomically diverse RCCs in proximity to the renal vein are invasion competent

Whole-exome profiles of spatially resolved TT samples did not reveal significant differences compared to PT in overall mutation burden, number or enrichment of driver mutations, or copy number variations (CNVs) (Fig. 1f and Supplementary Fig. 1e). 3p and 8p loss were prominent followed by 5q gain, 14q loss, 7q gain and 9p loss (Fig. 1f).

**Fig. 2:**
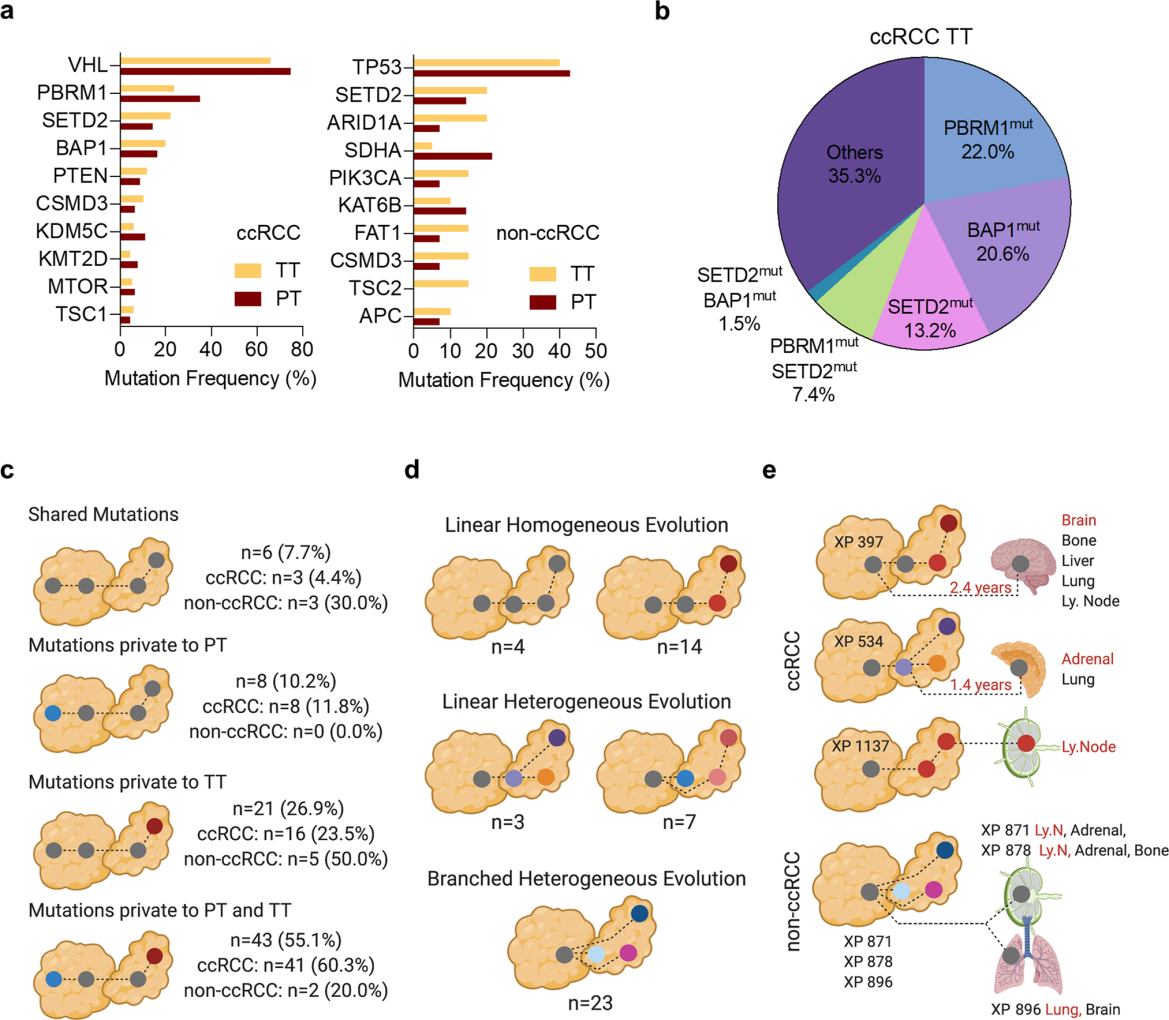
Oncogenomically diverse RCCs in geospatial proximity to the renal vein are invasion competent. **a** Observed mutational frequency of genes in RCC PT and TT. ccRCC (left) and non-ccRCC (right). **b** Composition of defined driver mutations in intravascular ccRCC TT samples. **c** Mutational patterns observed in matched PT and TT samples. **d** Observed clonal evolution patterns within TT from 51 RCC patients. **e** Clonal evolution patterns in paired PT, TT, and metastasis from six patients. Observed metastatic sites are highlighted (Red color names correspond to sequenced samples).

Mutation frequencies in driver genes were quite similar between PT and TT, and were comparable to other reports (Supplementary Fig. 2b). Compared to PT, no somatic alterations were overrepresented in TT. The majority of ccRCC driver mutations (*VHL, PBRM1, BAP1, mTOR, p53*, and *KDM5C)* were common to both PT and TT (Supplementary Fig. 2c). Overall mutation rates (in PT&TT) were as follows: *VHL* mutation (74.3%&65.9%); *PBRM1* (35.2%&23.7%); *SETD2* (14.3%&22.2%); *BAP1* (16.5%&20.0%); *PTEN* (8.8%&11.9%); and *CSMD3* (6.6%&10.4%) (Fig. 2a). *VHL* mutations co-occurred with *PBRM1* (28.5%&15.6%); *BAP1* (9.9%&10.4%); and *SETD2* (6.6%&11.9%) (Fig. 1f). As we reported earlier^20,21^, *BAP1* and *PBRM1* mutations were mutually exclusive (Fig. 1f).

Known ccRCC driver mutations were observed in approximately 65% of invasive TT. Based on driver mutations, TT could be classified into *PBRM1* mutant (22%); *BAP1* mutant (20.6%); *SETD2* mutant (13.2%); *PBRM1-SETD2* mutant (7.4%); and *BAP1-SETD2* mutant (1.5%) (Fig. 2b).

Non-ccRCC PT and TT (1 Chromophobe RCC, 1 Papillary RCC, 7 unclassified RCC, and 1 leiomyosarcoma) were also similar in somatic alterations and driver mutations (Supplementary Fig. 2d). 7p gain was observed across most non-ccRCC (Supplementary Fig. 1e). *TP53* mutations were observed in 40% (4/10) and *SETD2* mutations in 20% (2/10) of non-ccRCC TT (Supplementary Fig. 2e). *ARID1A* mutations were private to TT in 2/3 non-ccRCC patients, and so was a *TSC2* mutation (Supplementary Fig. 2f).

To assess TT evolution, we generated phylogenies using 332 defined driver mutations. Comparative analysis of matched PT and TT revealed four distinct patterns of clonal evolution (Fig. 2c). In more than 50% of cases (*n* = 43) different driver mutations were observed in the PT and matched TT suggesting divergent or independent evolution (Fig. 2c). Driver mutations private to either the PT or invasive TT were also observed in 10% and 27%, respectively. In 8% of the cases, PT and TT had the same mutational profile indicating co-evolution or clonal expansion. Detailed analyses of tumors with 2 or more TT samples (*n* = 51 patients) identified additional driver mutations in 92% of the TT cases (*n* = 47). Branched heterogenous evolution was observed in 45% (*n* = 23) of the cases. Linear homogenous evolution was observed in 35% (*n* = 18) while linear heterogenous evolution was observed in 20% (*n* = 10) (Fig. 2d).

These studies were further extended through analyses of metastases, which were available from 6 patients (29 multi-region samples from 3 ccRCC and 3 non-ccRCC patients). No significant differences in mutational frequencies or number of driver mutations were observed between the TT and metastatic samples. In 2 ccRCC patients (XP534 and XP1137), metastases had mutational profiles similar to the TT suggesting that tumor cell dispersion was through the intravascular extension (Fig. 2e). Linear evolution was observed in one ccRCC patient with metastasis to a lymph node. Driver phylogenies of non-ccRCC patients with synchronous metastasis showed overall greater similarity between metastases and the PT, suggesting early dissemination and divergent evolution with respect to TT (Fig. 2e).

These observations show that PT with diverse oncogenomic status irrespective of their association with disease aggressiveness can invade and extend as TT. Although TT evolve and acquire additional mutations, we found no association between overall somatic mutation status in PT with invasion and outgrowth in the vasculature.

### Invasion driven by immediate early genes represents a reversible adaptive response

**Fig. 3:**
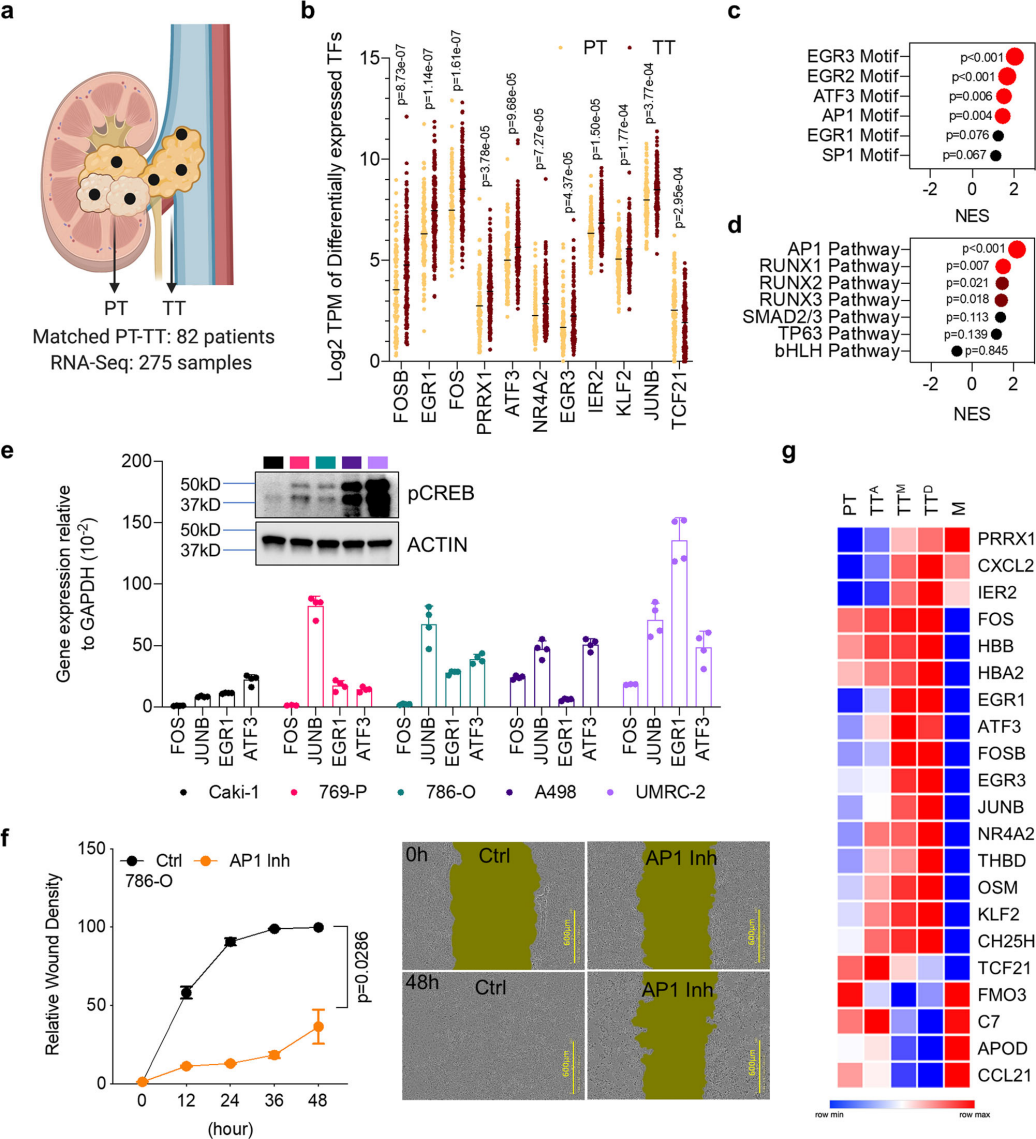
Invasion driven by immediate early genes represents a reversible adaptive response. **a** Illustration highlighting paired PT and TT samples used for transcriptomic analysis. **b** Differentially expressed transcription factors from biologically independent PT (*n* = 114) and TT (*n* = 161) patient tumor samples. Data are presented as scatter dot plots with lines at mean. Two-sided unpaired Student *t* test. **c**, **d** Motif enrichment (**c**) and signaling pathways (**d**) based on transcriptomic data from invasive TT. Nominal *p*-value from GSEA analysis <0.05 (brown color); <0.01 (red color). **e** qRT-PCR results showing expression of immediate early-response genes in ccRCC cell lines (*n* = 4 biologically independent samples). Data are presented as mean values ±SD. Western blot showing phospho-CREB status in ccRCC cell lines (inset). Source data are provided as a Source Data file. **f** Scratch wound healing assay of 786 ccRCC cells in the presence of AP-1 inhibitor T-5224 as indicated (*n* = 4 biologically independent samples). Data are mean values ±SEM. Two-tailed Mann–Whitney *U*-test. **g** Heat map showing changes in gene expression in PT, TT, and metastasis (M).

To identify gene programs associated with invasion, we analyzed transcriptomic profiles of invasive TT (Fig. 3a). We discovered a set of transcription factors that were differentially expressed between TT and PT (*p*-value < 0.05, FDRq < 0.2). The majority were immediate early genes (IEGs), including genes encoding components of the AP-1 complex (*FOSB, FOS*, and *JUNB*) as well as *EGR1, EGR3, IER2, ATF3*, and *NR4A2* (Fig. 3b). These genes are expressed as an adaptive response to external signals and are expressed in gliomas and prostate tumors (Supplementary Fig. 3a). Additionally, motif enrichment analysis revealed significant enrichment for genes with binding sites for AP-1 and EGR transcription factors in TT (Fig. 3c).

The TGF-beta responsive EMT determinant gene *PRRX1* was overexpressed in invasive TT (Fig. 3b). PRRX1 together with TWIST1, an EMT promoting transcription factor, drives cell invasion and dissemination^22–25^. Moreover, high expression of PRRX1 was associated with lower survival in renal cancer. In contrast, FOSB and EGR1 overexpression was favorable (Supplementary Fig. 3b–d). Pathway enrichment analysis of differentially expressed genes in TT showed enrichment for AP1 and TGF-beta driven responsive genes like RUNX (AP-1 interacting TF) and SMAD2/3 (Fig. 3d; Supplementary Fig. 3e; and Supplementary Data File. 1). These differentially expressed genes were also enriched in TGs, which were available from 8 patients, 8 PT-grafts, and 9 TT-grafts (Supplementary Fig. 4a).

Cellular plasticity driven by AP1 and EGR transcription factors is dependent on cyclic AMP response element binding protein (CREB)^26^. To test if increased CREB activity was associated with augmented expression of IEGs and invasion, we assayed pCREB levels in ccRCC cell lines. Caki-1 had the least pCREB activity, while 769-P and 786-O cells had moderate pCREB levels compared to A498 and UMRC-2 cells (Fig. 3e). Expression of IEGs strongly correlated with pCREB status in these cells (Fig. 3e; Source Data file. 1). Pharmacological inhibition of c-Fos/AP-1 using T-5224 reduced migration of ccRCC cell lines but did not affect cell proliferation (Fig. 3f and Supplementary Fig. 4b–d)^27,28^. Similar results were observed with siRNA knockdown of the AP1 transcription factor JUNB (Supplementary Fig. 4e–i).

A closer look at differentially expressed genes in TT identified several key factors. Kruppel like factor 2 (*KLF2*) and ß-globin genes (*HBB* and *HBA2*), which are induced in response to fluid shear stress and maintain cellular redox, were overexpressed in TT^29,30^. Likewise, expression of *CXCL2*, a cytokine implicated in migration, and *THBD*, which interferes with blood clotting, was increased in TT (Supplementary Fig. 4j).

As intravascular tumor cells extravasate and colonize distal organs, the IEG driven adaptive response is likely to be diminished. We tested this notion by assessing the expression of IEGs in metastatic lesions. Relative to the intravascular outgrowth, there was a significant reversal in expression of IEGs in metastatic lesions (M). The expression of ß-globin and clotting blocking factors enriched in TT was also attenuated in metastases (Fig. 3g). Next, we examined transcriptomic profiles of matched TT and M samples. EMT and TGF-b signaling was enriched in metastatic samples as expected while metabolic gene sets were enriched in TT compared to matched M clones (Supplementary Fig. 5a, b).

Taken together, these data implicate CREB/AP-1 driven IEG expression in TT cell migration as a reversible adaptive response to fluid shear stress in the vasculature.

### Identification of determinants of metastatic competence

**Fig. 4:**
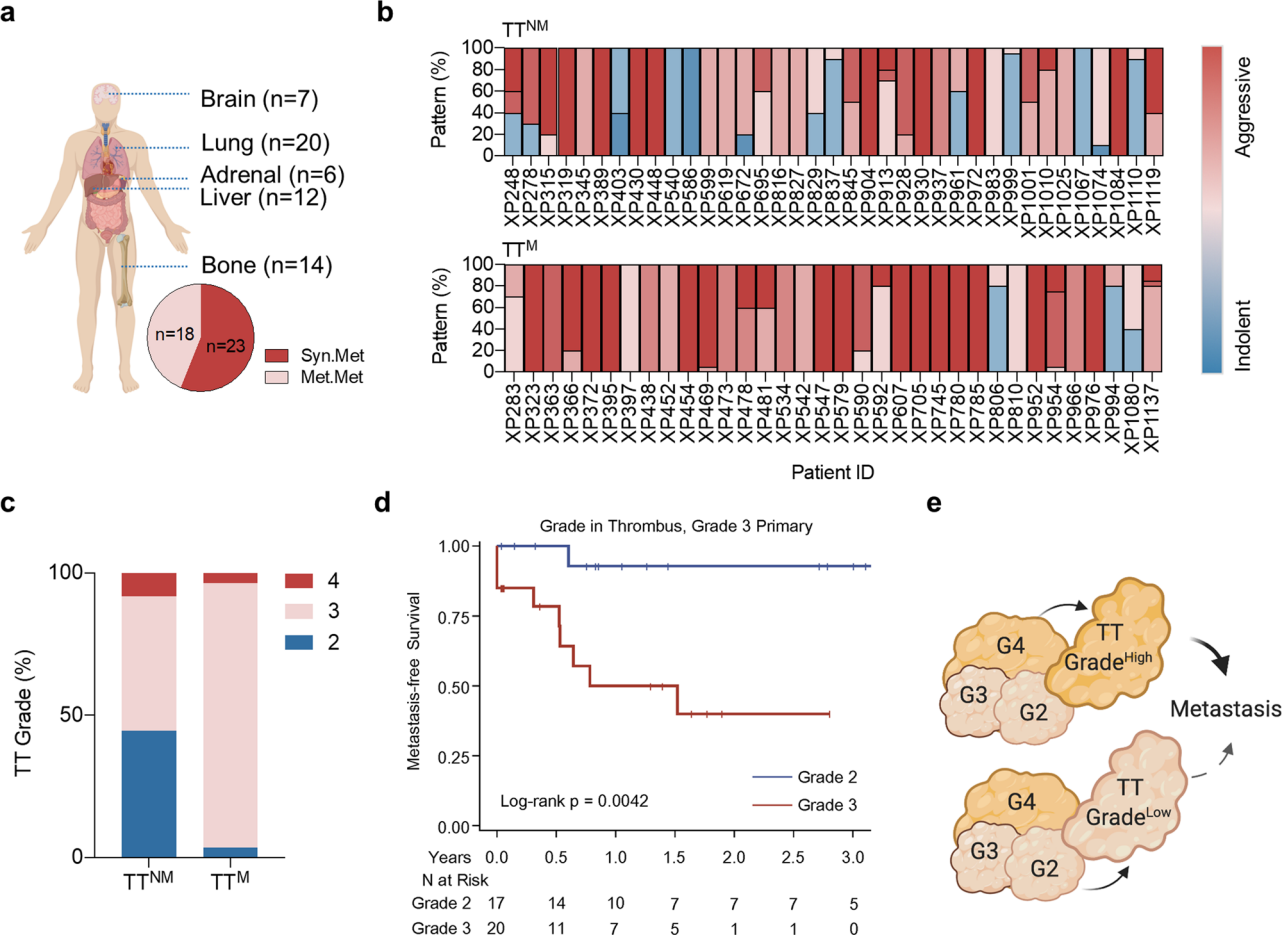
Tumor thrombus grade predicts metastatic outcome. **a** Metastatic sites in RCC patients with TT in this cohort. **b** Tissue architectural patterns are for TT. **c** Grade distribution in TT^M^ compared to TT^NM^. **d** Metastasis-free survival of patients with grade 3 PT classified according to TT grade. Kaplan–Meier (log rank) test. **e** Illustration highlighting higher metastatic incidence in RCC patients with high TT grade.

To delineate the relationship between invasion and metastatic competence, next we performed comparative analyses between patients with TT that metastasized and those that did not. At a median follow-up of 3 years, ~50% of patients in our cohort (*n* = 41) developed metastases (Fig. 4a, Supplementary Fig. 6a; and Supplementary Table. 1). Among the patients with metastasis (TT^M^), 23 presented with synchronous metastases and 18 developed metachronous metastases. Metastases mostly involved the lungs followed by bone and liver (Fig. 4a), a pattern which is characteristic of RCC. Forty-two patients with TT did not metastasize (TT^NM^). These two cohorts are remarkably different as illustrated also by Kaplan–Meier analyses, which showed substantial differences in overall survival (HR 4.29; 95% CI:1.24–14.82; *p* = 0.01) (Supplementary Fig. 6b). We systematically examined clinicopathological features, mutations, DNA copy number alterations and gene expression across the TT^M^ and TT^NM^ cohorts. However, no significant differences in somatic alterations were identified between TT^M^ and TT^NM^ (Supplementary Fig. 6c).

From a clinicopathological standpoint, we evaluated the impact of tumor size, focality, surgical margins, stage, tumor grade, sarcomatoid differentiation, tumor necrosis, invasion into the kidney parenchyma (non-intravascular) and lymph node involvement. These analyses revealed an association between TT^M^ with stage at presentation, tumor grade, sarcomatoid differentiation, tumor necrosis, kidney parenchyma invasion and lymph node involvement (Supplementary Table. 3). In addition, aggressive tissue architectural patterns were also enriched in TT^M^ relative to TT^NM^ (Fig. 4b) and in metastasis from paired ccRCC patients (Supplementary Fig. 6d).

To identify the most critical variables, we performed a Multivariate Cox proportional hazards analysis using time to metastasis. Unsurprisingly, these analyses showed that stage, sarcomatoid differentiation, and lymph node involvement were independent predictors of time to metastasis. Provocatively, however, grade in the TT but not overall tumor grade was also predictive. Furthermore, as determined by the hazard ratio, TT grade was the strongest predictor (HR, 6.42; *p* = 0.003). We observed higher tumor grade in TT^M^ relative to TT^NM^ (Fig. 4c). We focused on patients with grade 3 PT, which accounted for 52.1% in our cohort. Among these patients, 20 had TT that were grade 3 and 17 had TT that were grade 2. Interestingly, despite similarly high grade in the PT, patients with a TT of lower grade had significantly better metastasis-free survival than those with grade 3 TT (Fig. 4d).

Extending our previous observations that the most aggressive clone with the highest grade does not always seed the TT, these data show that the grade of the clone seeding the TT is a more important determinant of metastatic competency than overall tumor grade (Fig. 4e).

### Inflammatory immune contexture associated with metastatic competence

**Fig. 5:**
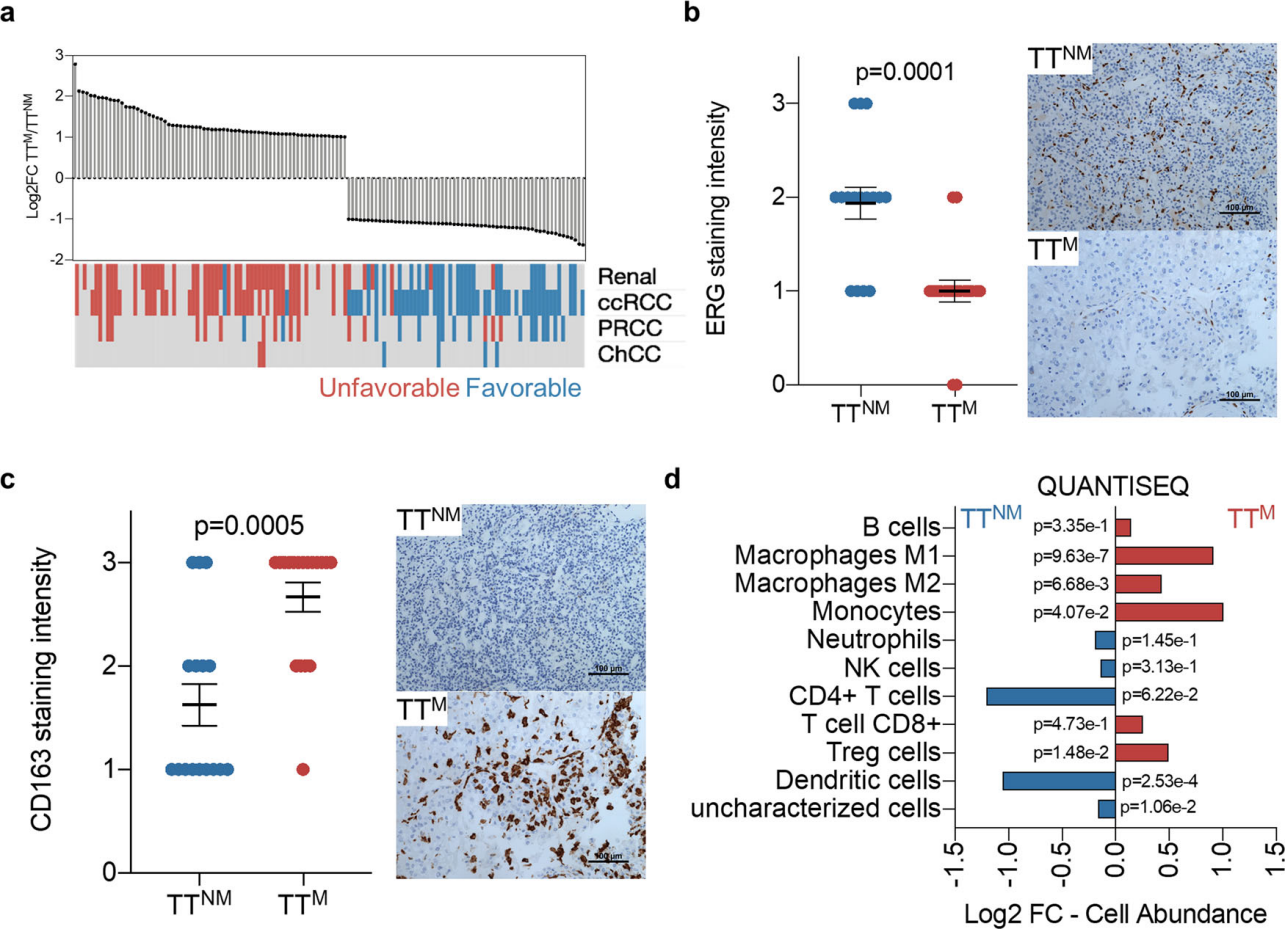
Stromal composition associated with metastatic tumor thrombus. **a** Differentially expressed genes between TT^NM^ and TT^M^ and their prognostic power based on human protein atlas. **b** Quantification and representative immunohistochemistry images of endothelial marker ERG in TT^NM^ and TT^M^ sections (*n* = 20 patients, 36 samples). **c** Quantification and representative immunohistochemistry images of macrophage marker CD163 in TT^NM^ and TT^M^ sections (*n* = 20 patients, 36 samples). Data are presented as scatter dot plots and lines at mean values ±SEM; two-tailed Mann-Whitney *U*-test (**b**, **c**). **d** TIMER analysis using a QUANTIFSEQ deconvolution-based approach revealing differences in stromal composition from biologically independent TT^NM^ (*n* = 78) and TT^M^ (*n* = 82) patient (tumor samples). Two-sided unpaired Student *t* test.

Next, we compared transcriptomic profiles of TT^M^ and TT^NM^. We identified 70 genes overexpressed and 61 under expressed in TT^M^ relative to TT^NM^ (Fig. 5a). These studies were complemented with studies of publicly available datasets where we hypothesized that genes upregulated in TT^M^ would be associated with poor prognosis. This was the case among different kidney cancer datasets, including for nccRCC histologies, as well as in other tumor types (Fig. 5a and Supplementary Fig. 7a, b).

Gene ontology analysis of TT^M^ genes identified leukocyte homeostasis and attenuated angiogenesis (Supplementary Fig. 7c). Gene set enrichment analysis revealed an increase in inflammation and mTOR signaling in TT^M^ (Supplementary Fig. 7d). Overall, attenuated expression of genes associated with angiogenesis and increased chemokine/cytokine expression was observed in TT^M^ (Supplementary Fig. 7e).

We validated these observations by immunohistochemistry (IHC) studies on flanking sections of TT^M^ and TT^NM^ samples used for gene expression analyses. As determined by ERG staining, endothelial cells were decreased in TT^M^ relative to TT^NM^ (Fig. 5b). A significant increase in pro-tumorigenic M2 macrophages (CD163^+^) was observed in the flanking sections of TT^M^ (Fig. 5c). In contrast, no significant differences in PD-L1 staining were observed (Supplementary Fig. 7f).

To further characterize differences in stromal composition between TT^M^ and TT^NM^, we deployed an empirically derived gene signature of the RCC tumor microenvironment (eTME)^31^. Supporting our findings, eTME analyses revealed an enrichment of macrophages and a reduction in endothelial cell markers in TT^M^ (Supplementary Fig. 7g). Monocytes and regulatory T cells were enriched, and NK cell markers were reduced in TT^M^. Similar observations were made with QUANTISEQ, EPIC, MCPCOUNTER, and Tumor IMmune Estimation Resource (TIMER)^32,33^ (Fig. 5d and Supplementary Fig. 7h–j). Macrophages and dendritic cell associated genes from QUANTISEQ are listed (Supplementary Fig. 7k–m). We are referring to our findings in Fig. 4d where higher TT grade is associated with metastatic outcome.  Likewise, inflammation and immune signatures are high in TT grade3/4 compared to TT grade 2. Analogous to our observations with TT^M^, TT grade 3–4 tumors were enriched for inflammation and immune signatures and deprived of angiogenesis related gene sets (Supplementary Fig. 8a, b).

### Augmented mTORC1 signaling determines metastatic potential

**Fig. 6:**
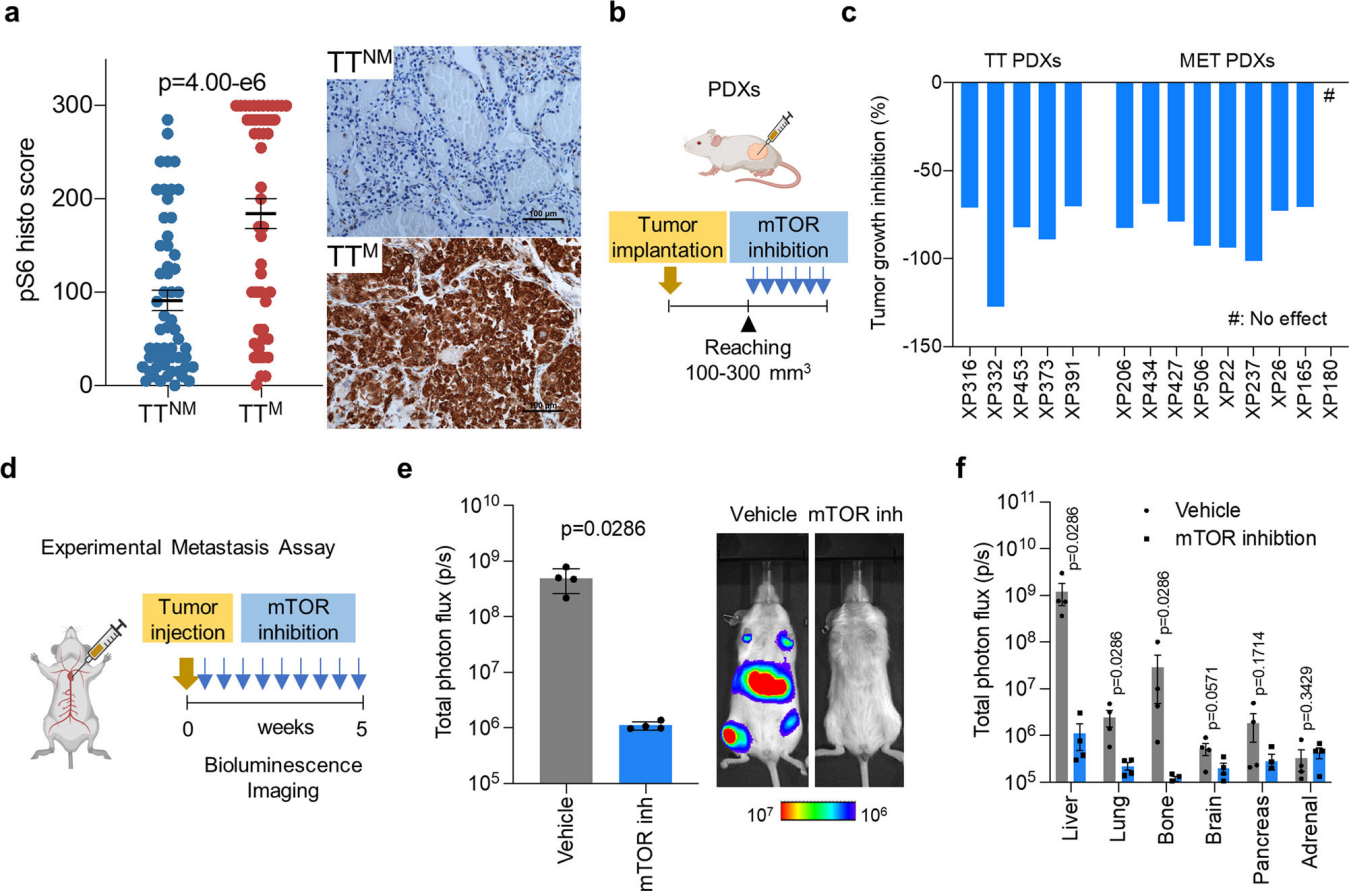
mTOR signaling is induced in metastatic tumor thrombus. **a** Quantification (left) and representative immunohistochemistry images (right) of phospho-S6 in TT^NM^ and TT^M^ (*n* = 65 patients, 81 samples). Data are presented as scatter dot plots and lines are at mean values ±SEM. Two-sided unpaired Student *t* test. **b** Schematic highlighting experimental protocol to inhibit mTOR in RCC patient-derived xenografts (PDXs). **c** Tumor growth inhibition rates of TT- and metastasis-derived PDX lines by mTOR inhibitor sirolimus (3–5 mice for each PDX). **d** Schematic of experimental protocol to inhibit mTOR in experimental metastasis assay. **e**, **f** Luciferase transduced 786-O ccRCC cells were intracardially injected and metastatic incidence was tracked by whole-body bioluminescent imaging (BLI: Total photon flux (p/s)) in mice treated with either vehicle or everolimus daily by oral gavage. BLI quantification and representative images of whole-body are shown (**e**). Quantification of metastatic burden in organs by ex vivo imaging (**f**). *n* = 4 mice each group. Data are presented as mean ±  SD. Two-tailed Mann–Whitney *U*-test.

To further evaluate whether mTOR (referring to mTOR complex 1, or mTORC1) was differentially activated between TT^M^ and TT^NM^, we performed immunohistochemical analyses. p-S6, a broadly used marker of mTORC1 activity, was significantly higher in TT^M^ (compared to TT^NM^). p-S6 was also increased in high grade TT (compared to low-grade TT) (Fig. 6a and Supplementary Fig. 9a). The mTOR gene signature was also enriched in TT compared to PT and M (Supplementary Fig. 9b, c) To evaluate the functional consequences of mTOR activation, we tested the impact of mTORC1 inhibition in PDX models. PDX models were generated from 5 TT^M^ and 9 metastases (XP373 and XP453 from this cohort and 12 additional ones). Mice bearing size-matched tumors (*n* = 3–5) were administered sirolimus, an allosteric mTORC1 inhibitor, and tumor progression was monitored compared to vehicle controls (*n* = 3–5) (Fig. 6b). 13/14 PDXs were sensitive to sirolimus resulting in a median tumor growth inhibition of 80.5% (Fig. 6c and Supplementary Fig. 9d, e).

Next, we assessed the impact of mTOR inhibition on metastasis (Fig. 6d). For these experiments, mice were injected intracardially with 786-O ccRCC cells transduced with GFP-luciferase to monitor metastatic colonization. Post injection, mice were randomized into two groups and treated with either vehicle or everolimus (a sirolimus analogue). Metastatic incidence was tracked using bioluminescent imaging. We observed a significant reduction in total body photon flux in mice treated with the mTOR inhibitor (Fig. 6e). Five weeks post-injection, mice were  euthanised. Ex vivo imaging and luciferase staining on sections showed a markedly reduced incidence of liver, lung, and bone metastases (Fig. 6f and Supplementary Fig. 9f–i). Overall, these data show that targeted inhibition of mTOR signaling in cancer cells impedes tumor progression and metastatic outbreaks.

## Discussion

Intratumor heterogeneity and sub-clonal evolution is characteristic of RCC^34–37^. Yet, metastases are more homogenous, and harbor fewer driver mutations and somatic alterations than PT, indicating that an evolutionary bottleneck process governs metastatic colonization^35,38^. The acquisition of invasion determinants is considered to be such a bottleneck^39,40^. However not all patients with invasive tumors develop metastases.

**Fig. 7:**
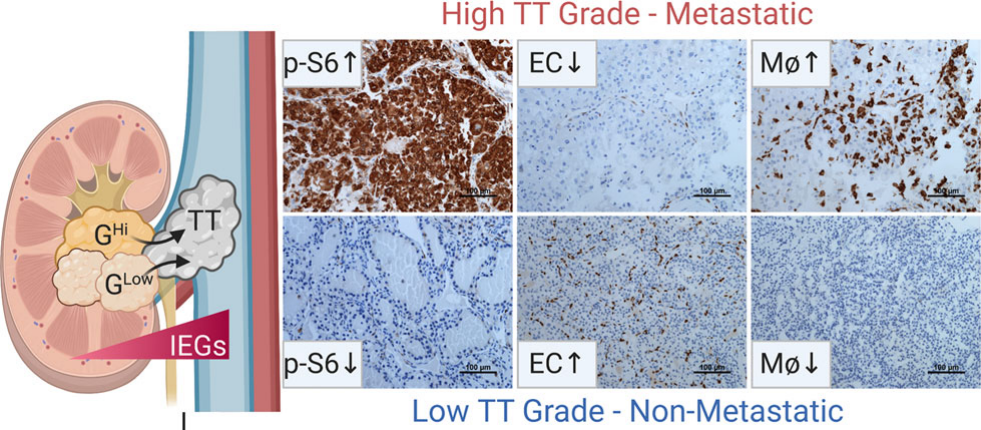
Determinants of invasion and metastatic competence in invasive intravascular RCC. Model highlighting key findings from the study. IEG overexpression in TT was based on gene expression analysis of biologically independent PT (*n* = 114) and TT (*n* = 161) samples. Tumor grade was analyzed using matched PT and TT from 71 ccRCC patients. Representative IHC images shown for p-S6 (*n* = 65 patients, 81 samples), Endothelial Cells (EC, anti-ERG, *n* = 20 patients, 36 samples), and Macrophages (Mϕ, anti-CD163 *n* = 20 patients, 36 samples).

Using a patient cohort assembled over a decade, we present studies to delineate invasion and metastatic competence employing paradigmatic RCC tumors with TT. We show that TT can be seeded by PT clones with diverse oncogenomic status (see model in Fig. 7). While *BAP1, SETD2, TSC1/2, TP53* mutations are associated with high tumor grade, disease aggressiveness, and poor survival^41–43^, we did not observe an enrichment of such mutations in TT. Both high- and low-grade clones were present in TT. These data support a contrasting model where intravascular invasion represents an opportunistic adaptive trait acquired by tumor clones in proximity to the renal vein segmental branches. Accordingly, invasion and cancer cell dispersion could be an early or late event depending on PT proximity to vasculature^44,45^.

Analyzing clearly defined invasive intravascular RCC tumor extensions revealed enrichment for a cellular plasticity program driven by immediate early genes (IEGs). Our study suggest that this IEG program represents an adaptive and transient response to fluid sheer stress during intravascular invasion^46,47^. Analyses of paired PT, TT and metastatic samples, show that the majority of genes enriched in TT were attenuated in metastatic lesions. This is in agreement with the transient nature of the invasion program and a lack of fixed irreversible somatic alterations dictating invasion. Only a subset of the differentially expressed genes in TT were retained in metastatic lesions. This subset included *PRRX1*, *CXCL*2 and *IER2*, and is consistent with their role in other tumor types^22,25,48,49^, where they have been implicated in metastasis. Understanding the downstream effectors of IEGs may provide mechanistic insights and strategies to limit RCC invasion and metastases.

Interestingly, despite gross invasion into the vasculature, we show that not all RCC TT tumors metastasize^50^. By comparing TT^M^ and TT^NM^, we identified metastatic competence determinants. We identified TT grade as an independent predictor of metastatic outcome. Accordingly, patients with lower grade TT had significantly reduced rates of metastases and better prognoses, than patients with higher grade TT irrespective of the grade of the PT. Whereas current prognosis/metastatic risk assessment tools consider the highest-grade area in the tumor^51,52^, our findings suggest that grade of TT (where applicable) is a more important determinant of metastatic potential and survival. These data are consistent with the notion that the invasive clone, rather than the most aggressive clone, is the primary driver of survival. Thus, determinants of invasion could be effective therapeutic targets to reduce metastases.

TT^M^ were characterized by augmented mTORC1 signaling. Increased mTORC1 activity may be driving the higher grade observed in TT^M^ compared to TT^NM^. Several findings support this notion. Grade is largely determined by the prominence of the nucleolus^18,53,54^, which is implicated in ribosome assembly, and protein translation, processes regulated by mTORC1. Furthermore, mTORC1 has been shown to regulate nucleolar size^55^. In addition, a correlation between higher grade and mTORC1 activation has been previously reported in RCC^56^. Perhaps most telling, disruption of *Tsc1* with consequent mTORC1 activation in *Vhl/Pbrm1-*genetically engineered mouse models is sufficient to increase tumor grade^57^. The importance of mTORC1 is highlighted through studies in animal models where mTORC1 inhibition reduced tumor growth and metastases. Accordingly, it is not surprising that mTORC1 inhibitors have also been proven to be effective for RCC patients, and two sirolimus analogues are approved by the FDA, temsirolimus and everolimus.

Beyond mTORC1 activation, TT^M^ were also characterized by pro-tumorigenic macrophages and reduced angiogenesis. Comprehensive dissection of the tumor and immune contexture within the TT using single cell sequencing and proteomic based analyses^58,59^ may provide mechanistic insight into how invading tumor cells achieve metastatic competence and evade immune surveillance^60^.

In summary, we report that invasion is an opportunistic trait attained by subclones with diverse oncogenomic status in geospatial proximity to the vasculature. We show that invasion is not necessarily driven by the most aggressive subclones in the tumor, and that the invasive subclones (rather than the most aggressive) appear to be the main determinants of metastatic competence. These results suggest that prognosis for RCC with TT is most accurately determined based on TT features, which has implications for clinical practice.

## Methods

### Patient selection

All patients that enrolled in the study provided informed consent allowing the use of discarded surgical samples for research purposes and genetic studies approved by UT Southwestern Institutional Review Board (The University of Texas Southwestern Medical Center Tissue Resource [STU 102010-051], Kidney Cancer New Pathway Discovery Project [STU 012011-190], and Comprehensive Database for Patients with Kidney Tumor [STU 022015-015]). Surgical schedules were screened weekly for RCC patients undergoing tumor excisions or biopsies. Inclusion criteria were largely based off imaging.

### Sample collection

Following surgical specimen acquisition, tumors were processed within 2 h. They were frozen for genomic studies or placed in 1% (vol/vol) penicillin-streptomycin (pen-strep) solution dissolved in phosphate-buffered saline for implantation in mice. For mouse transplantation, tumors were cut into 8 to 27 mm^3^ fragments and implanted orthotopically in the kidney of mice.

### Nucleic acid isolation from tissues

DNA and RNA were collected simultaneously as described before^19^ using AllPrep DNA/RNA mini kit (Qiagen). Tumor tissues were placed in RNAse-free 1.5 ml tubes with RLT buffer and homogenized with pestles. Debris was cleared with QIAshreder (Qiagen). Lysates were loaded on a DNA spin column and flow-through was collected for RNA isolation. After washing, DNA was collected in 10 mM Tris-Cl, pH 8.5 buffer. RNAs were isolated from the flow-through using TRIzol™ Reagent (Invitrogen) and RNA pellets were dissolved by 10 mM Tris-Cl, pH 8.5 buffer.

### Whole-exome sequencing

DNA was extracted from tumor tissue with adequate quality and submitted to Genentech for WES. Sequencing was performed using the HiSeq2500 platform (Illumina) to generate 2 × 75-bp paired-end data. For mutation calling, we used the QBRC mutation calling pipeline (https://github.com/Somatic-pipeline/Somatic-pipeline)^61^. Exome-seq reads were aligned to the human reference genome hg38 by BWA-MEM (version > =0.7.15). QC was done by fastqc. Picard (2.20.0) was used to add read group information and Sambamba (0.8.0) was used to mark PCR duplicates. The GATK toolkit (4.0) was used to perform base quality score recalibration and local realignment around Indels. MuTect (1.1.6), VarScan (2.4.2), Shimmer (0.2), SpeedSeq (0.1.2), Manta (version ≥1.4.0), Strelka (version ≥2.8.3), and Lofreq (version ≥2.1.3) were used to call SNPs and Indels. A mutation that was repeatedly called by any three of these programs was retained. We carried out somatic copy number variation (CNV) analyses using CNVkit (0.9.4) with default parameters on paired tumor-normal sequencing data. CNVkit uses both on- and off-target sequencing reads to calculate log2 copy ratios across the genome for each sample and improves accuracy in copy number calling by applying a series of corrections. Arm gain or loss was called when >50% of the chromosome exhibited copy number gain or loss. In order to have a chromosome arm level estimate of copy number (ranging from 0 to 4; normal would be 2), we calculated the weighted average of copy numbers from each gene within the target chromosome arm. The weights of each gene were derived from the ‘Bin-level Log2 ratios’ outputted by the CNVkit [https://github.com/etal/cnvkit], and the gene level copy numbers were calculated using the QBRC Somatic Pipelin5. Loss status was called if the chromosomal arm level copy number was ≤1 and gain was called if the chromosomal arm level copy number was > =3.

### RNA sequencing

TruSeq RNA Sample Preparation kit (Illumina) was used to prepare RNA-seq libraries. The libraries were multiplexed and sequenced using HiSeq2500 platform to obtain ~100 million paired-end (2 × 75-bp) reads per sample on average. FastQC was applied to control quality with the parameters “--extract --threads 48 -q.” RNA-seq reads were aligned to the human reference genome GRCh38 (hg38) utilizing STAR with the parameters “--runThreadN 48 --outSAMtype BAM Unsorted --outReandsUnmapped Fastx.” featureCounts19 with parameters “--primary -O -t exon -g transcript_id -s 0 -T 48 --largestOverlap --minOverlap 3 --ignoreDup -p -P -B -C” was then used to get expression level of genes. The human genome annotation file was downloaded from the University of California Santa Cruz (UCSC) table browser in the RefSeq Gene track. The file was employed by featureCounts (1.6.0). Read per kilobase million (RPKM) values were determined from read counts of genes. RPKM values were then transformed by log2. Next Generation sequencing data was analyzed using Morpheus (Broad Institute) and R software (version 3.6.0).

### Animal experiments

Animal experiments were performed in accordance with the *Guide for Care and Use of Laboratory Animals* approved by the UT Southwestern Institutional Animal Care and Use Committee (IACUC). Tumor fragments from stably engrafted tumorgrafts were implanted subcutaneously in 4–6-weeks-old NOD-SCID mice. Tumors were routinely reviewed by a dedicated genitourinary pathologist (P.K.) to select areas with viable tumor cells for study. When tumor volumes reached ~100–300 mm^3^, mice were allocated into different treatment groups on the basis of following criteria: tumor volume, growth rate, and mouse weight^62,63^. Vehicle (10% EtOH, 30% PEG400, 60% MCT (0.5% methyl cellulose, 0.5% Tween 80 (aq)) was administered by gavage every 12 h. Sirolimus (LC Laboratories) was administered intraperitoneally every 48 h at 0.5 mg/kg in 5% PEG400, 5% Tween80 in D5W. Mouse weight and tumor volume were assessed three times per week.

NOD.CB17-*Prkdc*^*scid*^ *Il2rg*^*tm1Wjl*^/SzJ (NSG) female mice from 4–6 weeks of age were used for experimental metastasis assay. Luciferase labeled 2.0 × 10^5^ cells were resuspended in 100 μl 1X PBS and intracardially injected into the right ventricle. Metastatic tumors were detected by non-invasive bioluminescence imaging using an IVIS spectrum. Bioluminescence images were quantified using ROI tool in living Image software (version 4.4). DMSO or Everolimus (5 mg/kg) in 1% methylcellulose solution was given by oral gavage daily post-intracardiac injection.

### Morphologic assessment and immunohistochemistry

Tissue samples for analysis were obtained from the primary renal tumor, tumor thrombus, metastatic sites, and matched normal tissue. Both fresh frozen and formalin-fixed, paraffin-embedded (FFPE) samples were used. The morphologic architecture, cytologic pattern, and tumor microenvironment in each sample were centrally reviewed and characterized by two genitourinary pathologists (PK and SC) after obtaining hematoxylin/eosin (H&E) stained slides. Flanking sections of FF tissue used to extract DNA/RNA were evaluated as indicated. Morphologic patterns were characterized by a classification system recently developed by our group^17^. To define morphologic patterns present in ccRCC, we examined the spatial architecture, cytologic features, and the tumor microenvironment (TME) within different regions of the tumor and tumor thrombus. Tumor samples were investigated using a Nikon Eclipse E100-LED multihead magnifying lens (Nikon Instruments Inc.), and the whole range of morphologic components pictured in the whole case (not simply the high-grade regions) was classified. The grading delivered in the pathology report for each case was affirmed. The assessed rate (least of 5% was expected) of each architectural pattern and the presence or absence of the cytologic and TME characteristic (even when focal) were arranged for every tumor manually by 2 specialists who were blinded to the diagnosis of patient finding and clinical data.

The WHO/ISUP^4^ grade were determined on routine hematoxylin and eosin–stained tumor sections by two pathologists blinded to clinical data. Confirmation of ccRCC diagnosis and grade was performed for each case (as assigned by a group of specialists in the GU pathology division) by two GU pathologists. In each case, both reviewers (an experienced uropathologist (P.K.) and a GU pathology fellow (S.C.) determined WHO/ISUP grading system for the thrombus independently. In cases with interobserver variations, slides were reevaluated, and grade was assigned. Before performing this review, both pathologists reviewed WHO/ISUP grading systems in 10 representative ccRCC cases to evaluate interrater agreement.

IHC staining for CD31 (1:100, Agilent technology, Cat# M082301-2), ERG (1:100, Biocare Medical, Cat# CM 421 A, C), CD163 (1:100, Biocare Medical, Cat# CM 353 AK, CK), and phosphorylated S6 ribosomal protein (Ser235/236) (1:100, Cell Signaling, Cat# 2211) was performed on FFPE tissue using Autostainer Link 48 (Dako). pS6 expression was quantified as a mean H-score, determined as the product of staining intensity (0–3) and extent of immunostaining (percentage of cells with staining). Appropriate positive and negative controls were used for each run and checked for validation.

### In vitro studies

Cell lines were obtained from ATCC and confirmed as mycoplasma free using Universal Mycoplasma Detection Kit (ATCC). A498, Caki-1 and UMRC-2 cells were provided by Qing Zhang, UT Southwestern. Cells were maintained in RPMI-1640 (769-P and 786-O) or DMEM (A498, Caki-1 and UMRC-2) supplemented with penicillin/streptomycin, l-glutamine, and 10% fetal bovine serum (FBS) at 37 °C in a humidified atmosphere with 5% CO_2_. *JUNB* siRNAs was obtained from Dharmacon. ccRCC cell lines were transfected using 25 mM siRNAs and DharmaFECT reagent (Dharmacon) for 24 h and knockdown efficiency was checked after 48 h of transfection by qRT-PCR.

### qRT-PCR

RNAs were collected from cultured ccRCC cell lines using RNeasy mini plus kit (Qiagen). cDNAs were generated from 1μg of total RNA using iScript™ cDNA Synthesis Kit (Bio-Rad). Quantitative RT-PCR was performed using SYBR Green Super mix (Bio-Rad) with specific primers and cDNAs. Specific primer sequences used are listed in Supplementary Table. 5.

### Western blot

Cultured ccRCC cell lines were washed with ice-cold Phosphate-buffered saline (PBS). RIPA buffer with cOmplete™, Mini Protease Inhibitor Cocktail (Roche) and PhosSTOP (Roche) was added to each 10 cm cell culture plate. Cells were scraped and incubated on ice for 15 min. Lysates were centrifuged for 10 min at 16,000×*g* and supernatants were collected into new tubes and protein concentration was measured using Pierce™ BCA Protein Assay Kit (Thermo). 10 μg of protein was loaded on a SDS-PAGE gel and lysates were transferred to a nitrocellulose membrane. Membranes were blocked for 1 h with 5% non-fat milk in TBST and incubated with primary antibodies overnight. Primary antibodies for Phospho-CREB (Ser133) (1:1000, Cell Signaling, Cat# 9198) and Beta Actin (1:25000, Abcam, Cat# ab49900) were used. After secondary antibody incubation with goat anti-rabbit IgG (H + L) HRP conjugated antibody (1:5000, Milipore, Cat# AP307P), the membranes were developed using chemiluminescence. Uncropped scans of the blots are supplied in Source Data file. 1.

### Scratch wound healing assay

After ccRCC cells reached confluency in 96-well tissue culture plates, IncuCyte Woundmaker (Essen BioScience) was used to create a wound in the cell monolayers. The AP1 inhibitor T-5224 was added as indicated. Cell migration was followed by IncuCyte every 4 h for 48 h. Cell migration was quantified using IncuCyte software.

### Gene Set Enrichment Analysis

Ranked Gene Set Enrichment Analysis (GSEA) was performed with software version 4.0.3. Default settings were used except for “No Collapse” and “Phenotype” permutation type to “Gene_set”. Defined lists from the Broad Institute were used: h.all.v.7.0.symbol.gmt [Hallmarks], c2.all.v.7.0.symbol.gmt [Curated], c3. all.v.7.0.symbol.gmt [motif], and c5.all.v.7.0.symbol.gmt [Gene Ontology]. Statistical criteria are described in results and figure legends.

### Clonal evolution pattern analysis

For each tumor, a matrix was generated based on WES results including frameshift substitutions, non-frameshift substitutions, stop gain, stop loss, splicing mutations, non-synonymous SNVs, and known mutations. 332 driver genes with mutations (Supplementary Table 6) were used to generate phylogenetic trees for each patient based on following rules: clones having the same mutation profiles were filled with same colors whereas clones with additional mutations were filled with different colors. Branching points were decided based on the presence of distinct mutations. The length of lines does not represent mutational similarity or duration to acquire mutations among clones.

### Quantification and statistical analysis

Data was analyzed and statistics were performed in Prism software v8.0 (Graphpad). Statistical significance was determined using a one-tailed or two-tailed Mann–Whitney *U* test or a Student *t* test. Kaplan–Meier (log rank) test was used for survival analysis. Fisher’s exact test was used to test associations between categorical characteristics and the metastasis status. Multivariate Cox proportional hazards model was used for multivariate analysis.

### Reporting summary

Further information on research design is available in the Nature Research Reporting Summary linked to this article.

## Supplementary information

Supplementary Information

Description of Additional Supplementary Files

Supplementary Data 1

Reporting Summary

## Data Availability

For patients explicitly consenting to depositing of genomic information in a public database, the raw WES/RNA-seq data is deposited in European Genome-phenome Archive (EGA) under the accession number EGAS00001005511 and EGAS00001005512. Data deposited in EGA database can be accessed based on data access agreement. All 83 patients consent for research. However, two patients did not want to release their genomic data for privacy. Access of processed genomic data including non-deposited data can be requested by contacting Alana Christie [Alana.christie@utsouthwestern.edu] and Data Access Committee [https://ega-archive.org/dacs/EGAC00001002272]. The user would have to abide by the same rules as outlined in the EGA data access agreement. The timeframe for response to requests is of 2 weeks. cBioPortal [https://www.cbioportal.org] was used to evaluate mutational frequencies of ccRCC patients from other studies (Datasets used: Kidney Renal Clear Cell Carcinoma (TCGA, Firehose legacy, [https://www.cbioportal.org/study/summary?id=kirc_tcga]), Kidney Renal Clear Cell Carcinoma (TCGA, PanCancer Atlas, [https://www.cbioportal.org/study/summary?id=kirc_tcga_pan_can_atlas_2018]), and Renal Clear Cell Carcinoma (UTokyo, Nat Genetics, [https://www.cbioportal.org/study/summary?id=ccrcc_utokyo_2013]). and Pearson correlation coeficients of differentially expressed genes were calculated (Dataset used: Kidney Renal Clear Cell Carcinoma (TCGA, Firehose Legacy, [https://www.cbioportal.org/study/summary?id=kirc_tcga]), Pediatric Wilms’ Tumor (TARGET, 2018, [https://www.cbioportal.org/study/summary?id=wt_target_2018_pub]), Prostate Adenocarcinoma (TCGA, Firehose Legacy, [https://www.cbioportal.org/study/summary?id=prad_tcga]), Breast Invasive Carcinoma (TCGA, Firehose Legacy, [https://www.cbioportal.org/study/summary?id=brca_tcga]), Kidney Renal Papillary Cell Carcinoma (TCGA, Firehose Legacy, [https://www.cbioportal.org/study/summary?id=kirp_tcga]), Brain Lower Grade Glioma (TCGA, Firehose Legacy, [https://www.cbioportal.org/study/summary?id=lgg_tcga]), Glioblastoma Multiforme (TCGA, Firehose Legacy, [https://www.cbioportal.org/study/summary?id=gbm_tcga]), Pancreatic Adenocarcinoma (TCGA, Firehose Legacy, [https://www.cbioportal.org/study/summary?id=paad_tcga]), Head and Neck Squamous Cell Carcionma (TCGA, Firehose Legacy, [https://www.cbioportal.org/study/summary?id=hnsc_tcga]), Kidney Chromophobe (TCGA, Firehose Legacy, [https://www.cbioportal.org/study/summary?id=kich_tcga]), Metastatic Prostate Adenocarcinoma (SU2C/PCF Dream Team, PNAS 2019, [https://www.cbioportal.org/study/summary?id=prad_su2c_2019]), Liver Hepatocellular Carcinoma (TCGA, Firehose Legacy, [https://www.cbioportal.org/study/summary?id=lihc_tcga]), Lung Adenocarcinoma (TCGA, Firehose Legacy, [https://www.cbioportal.org/study/summary?id=luad_tcga]), Skin Cutaneous Melanoma (TCGA, Firehose Legacy, [https://www.cbioportal.org/study/summary?id=skcm_tcga]), and Bladder Urothelial Carcinoma (TCGA, Firehose Legacy, [https://www.cbioportal.org/study/summary?id=blca_tcga]). The human protein atlas^64^ was used to obtain prognostic power of differentially expressed genes in TT (PRRX1, FOSB, and EGR1). Timer was used to analyze immune composition in TT^NM^ and TT^M^ ([http://timer.cistrome.org]). Source data are provided as Source Data file. Source data are provided with this paper.

## Source data

Source Data
