# Supplementary material for: Determinants of renal cell carcinoma invasion and metastatic competence
Source: Nat Commun. 2021 Oct 4;12:5760. doi: 10.1038/s41467-021-25918-4 (PMC8490399; doi:10.1038/s41467-021-25918-4)
Supplement: Supplementary file 1 — Supplementary Information [file 41467_2021_25918_MOESM1_ESM.pdf]

Determinants of Renal Cell Carcinoma Invasion and Metastatic Competence

Kangsan Kim[1,2], Qinbo Zhou[3], Alana Christie[4,5], Christina Stevens[4,5], Yuanqing Ma[4,5], Oreoluwa Onabolu[4,5], Suneetha Chintalapati[1], Tiffani Mckenzie[4,5], Vanina Toffessi Tcheuyap[4,5], Layton Woolford[4,5], He Zhang[6], Nirmish Singla[4,7], Pravat Kumar Parida[1,2], Mauricio Marquez-Palencia[1,2], Ivan Pedrosa[4,7,8], Vitaly Margulis[4,7], Arthur Sagalowsky[7], Zhiqun Xie[3,4,9], Tao Wang[3,4,9], Steffen Durinck[10], Zora Modrusan[10], Somasekar Seshagiri[10,11], Payal Kapur[1,4,*], James Brugarolas[4,5,*] and Srinivas Malladi[1,2,12,*]

[1]Department of Pathology, UT Southwestern Medical Center, Dallas, TX, 75390, USA
[2]Harold C. Simmons Comprehensive Cancer Center, UT Southwestern Medical Center, Dallas, TX, 75390, USA
[3]Quantitative Biomedical Research Center, Department of Population and Data Sciences, University of Texas Southwestern Medical Center, Dallas, TX, 75390, USA
[4]Kidney Cancer Program, Simmons Comprehensive Cancer Center, The University of Texas Southwestern Medical Center, Dallas, TX, 75390, USA
[5]Hematology-Oncology Division, Department of Internal Medicine, University of Texas Southwestern Medical Center, Dallas, TX, 75390, USA
[6]Bioinformatics Core Facility, The University of Texas Southwestern Medical Center, Dallas, TX, 75390, USA
[7]Department of Urology, The University of Texas Southwestern Medical Center, Dallas, TX, 75390, USA
[8]Department of Radiology, University of Texas Southwestern Medical Center, Dallas, TX, 75390, USA
[9]Center for the Genetics of Host Defense, University of Texas Southwestern Medical Center, Dallas, TX, 75390, USA
[10]Molecular Biology Department, Genentech, Inc., South San Francisco, CA, 94080, USA
[11]SciGenom Research Foundation, Bangalore, 560099, India.
[12]Lead Contact
*Correspondence: payal.kapur@utsouthwestern.edu (P.K), james.brugarolas@utsouthwestern.edu (J.B), and srinivas.malladi@utsouthwestern.edu (S.M)

Table of Contents

1. Supplementary Figures 1-9
2. Supplementary Tables 1-6

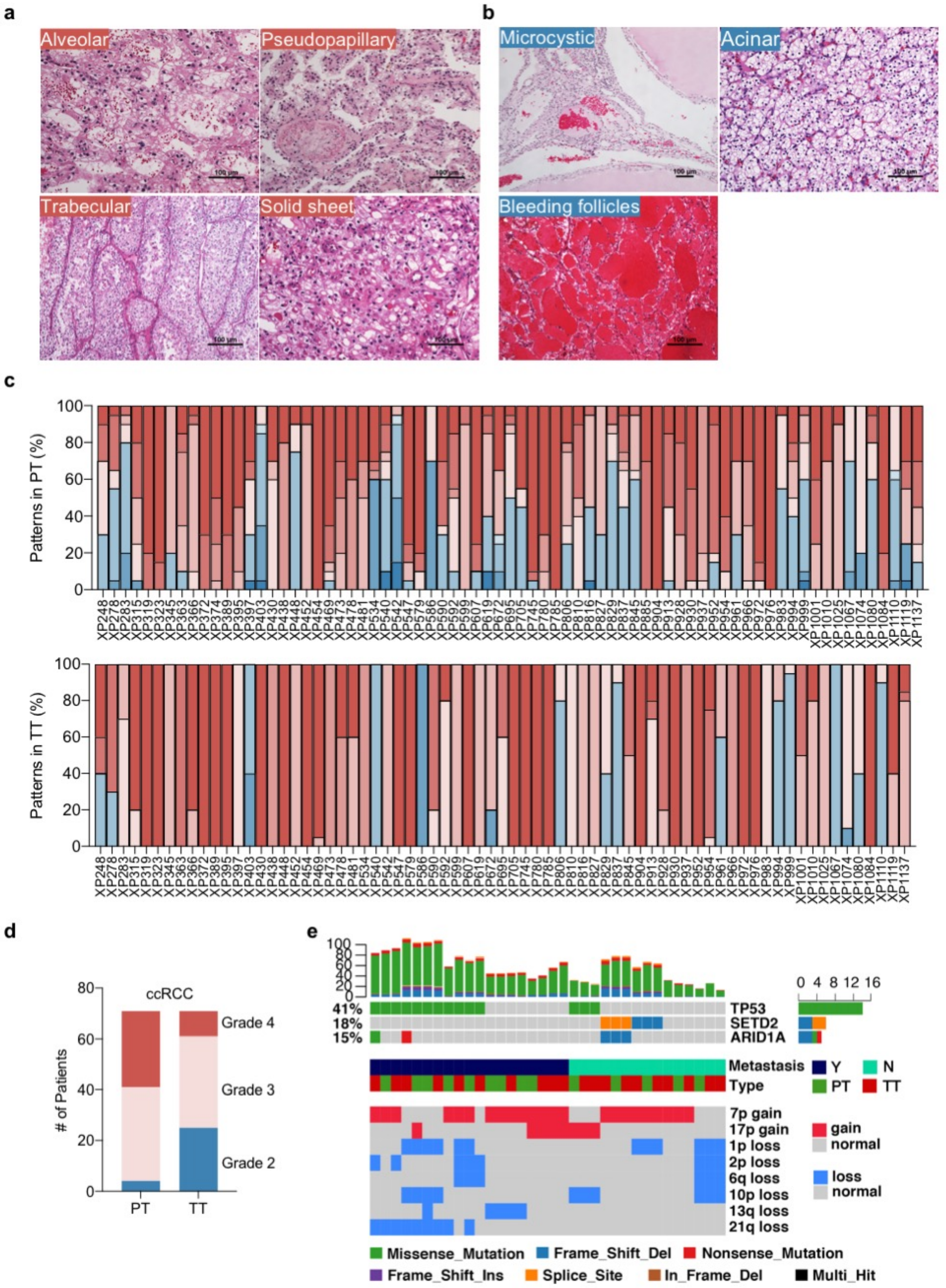

Supplementary Fig. 1: Architectural patterns and tumor grade in RCC patients with invasive intravascular tumor thrombus. **a**,**b,** Representative images of aggressive (**a**) and indolent (**b**) patterns in PT and TT from 71 ccRCC patients. **c,** Distribution of architectural patterns in PT and TT from 71 ccRCC patients (2 patients were excluded due to limited TT sample). **d,** Nuclear grade of PT and TT in 71 ccRCC patients. **e,** Overview of mutations, CNVs and clinical parameters from non-ccRCC patients.

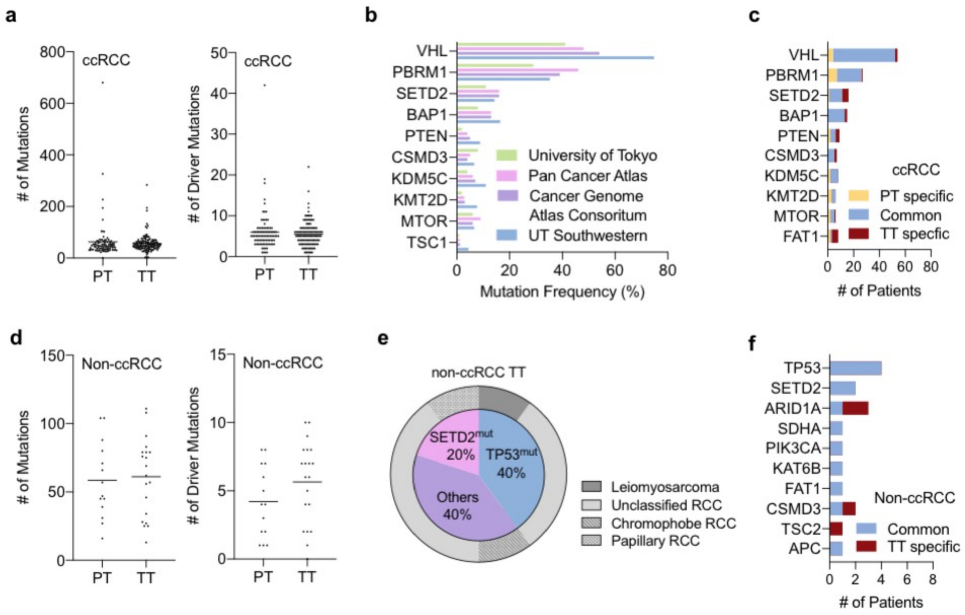

Supplementary Fig. 2: Oncogenomic profiles of tumor thrombus ccRCC and nccRCC cohorts. **a,** Total number and driver number of mutations of PT (n=91) and TT (n=135) in ccRCC samples. Data are presented as scatter dot plots and lines are at mean. **b,** Somatic mutational frequency in this cohort and other published ccRCC datasets. **c,** Somatic mutations according to commonality between PT and TT (common), private to PT (PT specific) or TT (TT specific) in ccRCC patients. **d,** Number of mutations and driver mutations of PT (n=14) and TT (n=20) in non-ccRCC patients. Data are presented as scatter dot plots and lines are at mean. **e**, Composition of defined driver mutations in intravascular non-ccRCC TT samples. **f,** Somatic mutations common in PT and TT (common), private to PT (PT specific) or TT (TT Specific) in non-ccRCC patients.

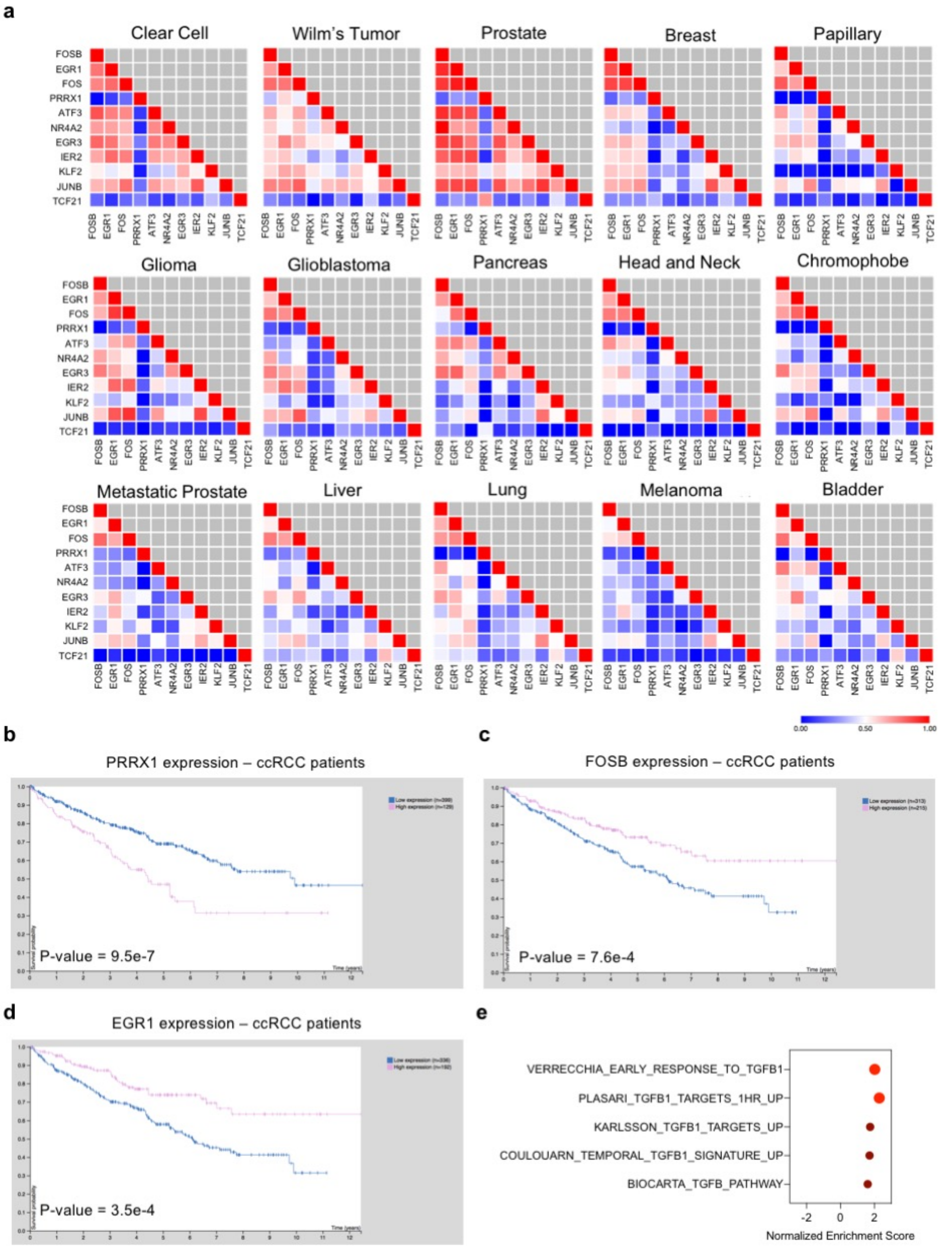

Supplementary Fig. 3: Genes differentially expressed in TT. **a,** Pearson correlation of differentially expressed genes in TT across various tumor types. **b-d,** Prognostic power of PRRX1 (**c**), FOSB (**d**), and EGR1 (**e**) expression in ccRCC. Images are available from Human Protein Atlas. [https://www.proteinatlas.org/ENSG00000116132-PRRX1/pathology/renal+cancer/KIRC], [https://www.proteinatlas.org/ENSG00000125740-FOSB/pathology/renal+cancer/KIRC], and [https://www.proteinatlas.org/ENSG00000120738-EGR1/pathology/renal+cancer/KIRC]. Kaplan-Meier (log rank) test. **e,** Gene set enrichment analysis showing enrichment for TGFβ related gene sets in TT compared to PT.

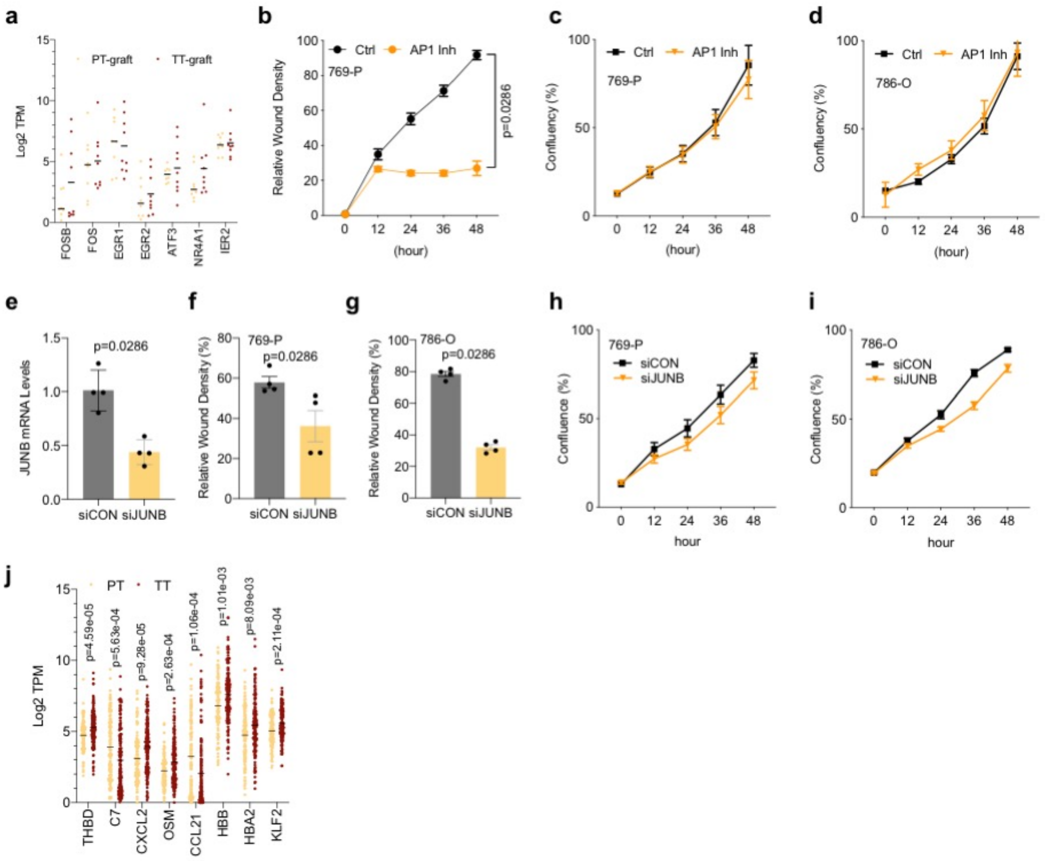

Supplementary Fig. 4: AP1 role in ccRCC migration. **a**, mRNA expression of IEGs in biologically independent PT-graft (n=8) and TT-graft (n=9) in mice. Data are presented as scatter dot plots and lines are at mean. **b,** Scratch wound healing assay results of c-Fos/AP-1 inhibitor T-5224 in 769-P cells. **c-d**, Proliferation assay results of c-Fos/AP-1 inhibitor T-5224 in 769-P (**c**) and 786-O (**d**) cell lines. **e**. Knockdown validation of *JUNB* siRNA. **f-i**. Scratch wound healing assay (**f,g**) and proliferation assay (**h,i**) under siRNA treatment. n=4 biologically independent samples. Data are presented as mean +/- SEM. Two-tailed Mann-Whitney *U*-test (**b-i**). **j,** Other differentially expressed genes from biologically independent PT (n=114) and TT (n=161). Data are presented as scatter dot plots and lines are at mean. Two-sided unpaired student t-test.

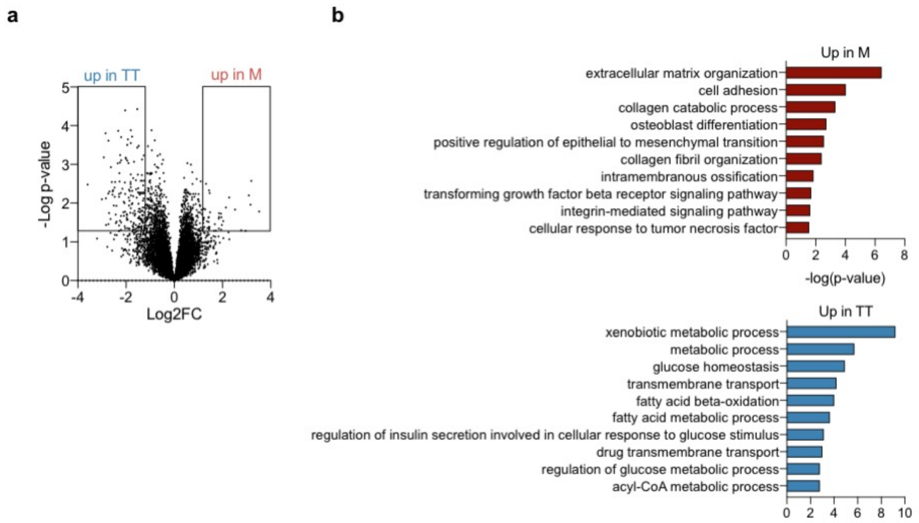

Supplementary Fig. 5: Enriched signatures in metastatic clones. **a,b**, Differentially expressed genes (**a**) and enriched gene signatures between TT and M (**b**). Two-sided unpaired student t-test (**a**). Nominal p-value from GSEA analysis (**b**).

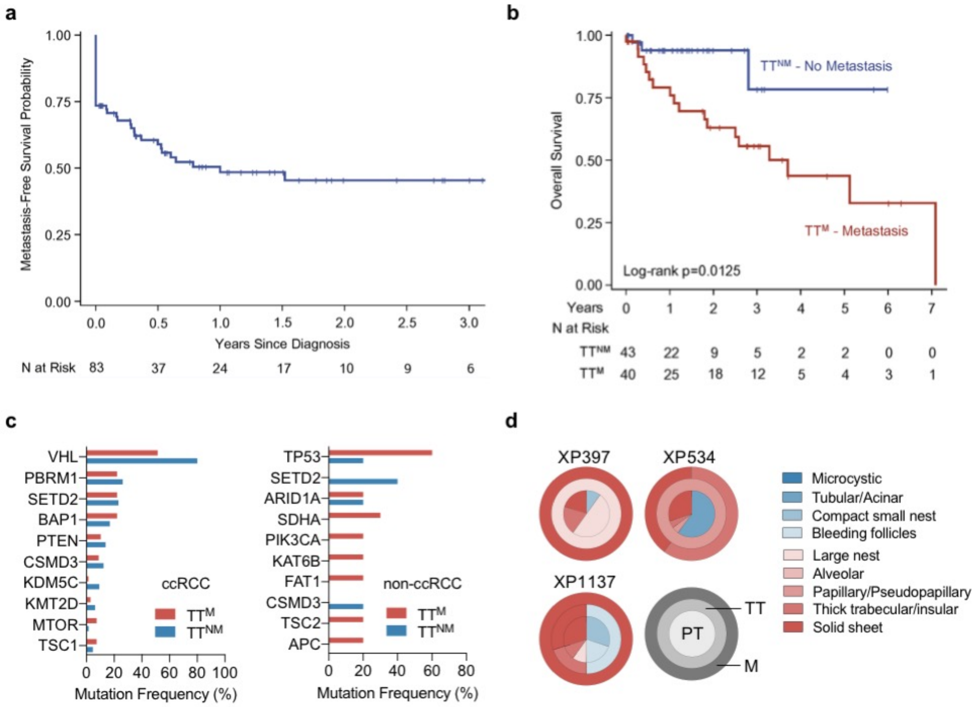

Supplementary Fig. 6: Traits associated with metastatic TT. **a,** Observed metastatic-free survival in RCC patients with invasive TT in this cohort. **b,** Overall survival of RCC patients with intravascular TT based on metastatic incidence. Kaplan-Meier (log rank) test. **c,** Somatic mutational frequency observed in non-metastatic tumor thrombus (TT^{NM}) and metastatic tumor thrombus (TT^{M}) in ccRCC (left) and non-ccRCC patients (right). **d,** Histologic architectural patterns of matched PT (inner most circle), TT (middle), and metastasis (outer circle) of 3 ccRCC patients.

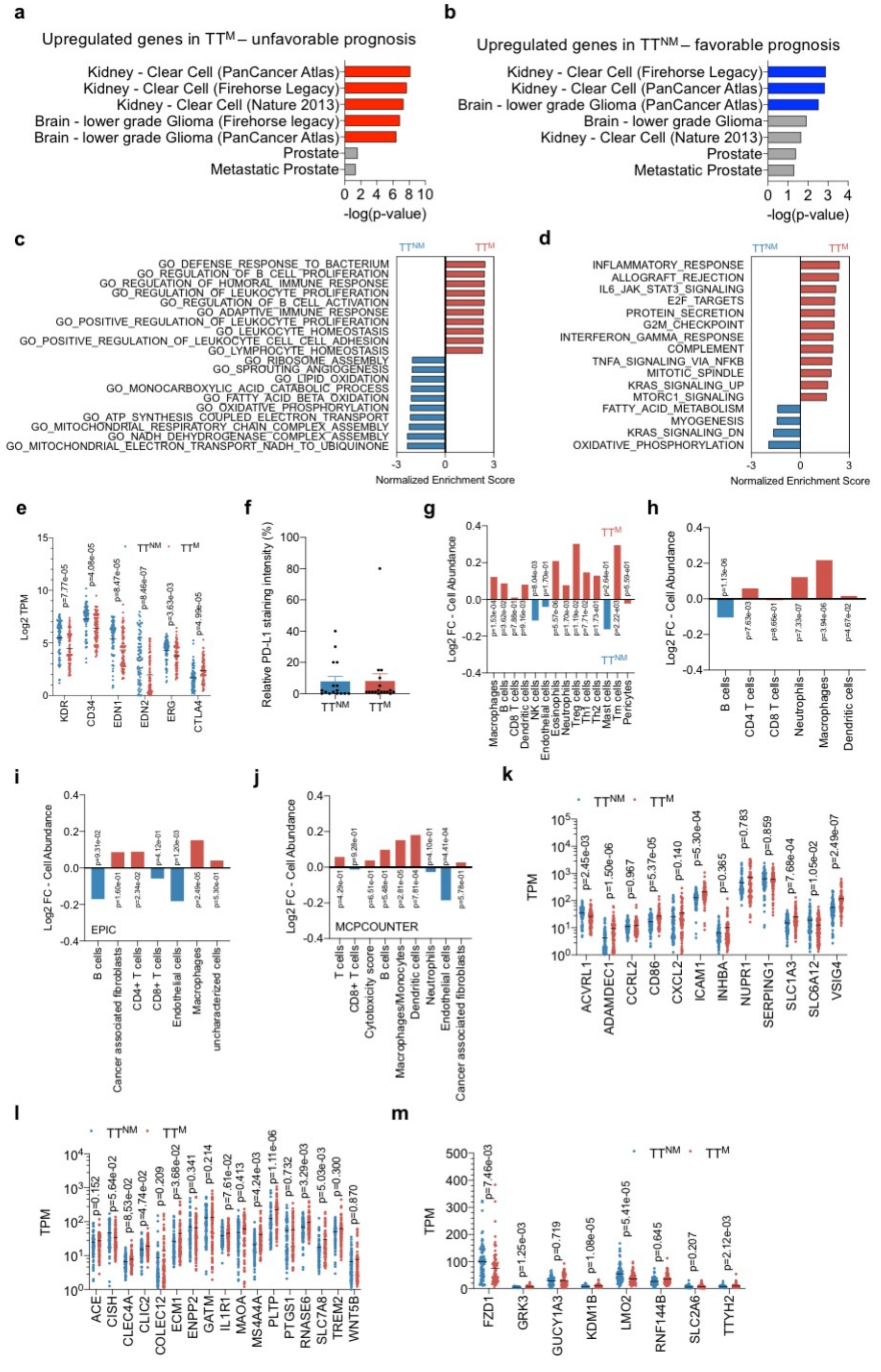

Supplementary Fig. 7: Altered stromal composition dictates metastatic incidence. **a-b,** Prognostic power of differentially expressed genes in metastatic TT (p-value - Kaplan-Meier (log rank) test from cBioportal). **c,** Enriched Gene Ontology (GO) gene sets in metastatic tumor thrombus compared to non-metastatic tumor thrombus. **d,** List of significantly altered hallmark gene sets (Nominal p-value < 0.01 from GSEA analysis) between TT^{NM} and TT^{M}. **e,** Gene expression of T-regulatory cell marker *CTLA4* and endothelial cell markers from biologically

independent TT^{NM} (n=78) and TT^{M} (n=82) patient tumor samples. Data are presented as scatter dot plots and lines are at mean. Two-sided unpaired student t-test. **f,** Quantification of immunohistochemistry images of PD-L1 using TT^{NM} (n=16, 10patients) and TT^{M} (n=17, 10 patients) slides. Data are presented as mean +/- SEM. **g-j,** Database deconvolution-based approach eTME analysis (**g**), TIMER (**h**), EPIC (**i**), and MCPCOUNTER (**j**) to identify stromal components in TT^{NM} and TT^{M} from biologically independent TT^{NM} (n=78) and TT^{M} (n=82). Two-sided unpaired student t-test. **k-m**, TPM gene expression values used for QUANTISEQ database analysis of M1 macrophages (**k**), M2 macrophages (**l**), and dendritic cells (**m**) from biologically independent TT^{NM} (n=78) and TT^{M} (n=82). Data are presented as scatter dot plots and lines are at mean. Two-sided unpaired student t-test.

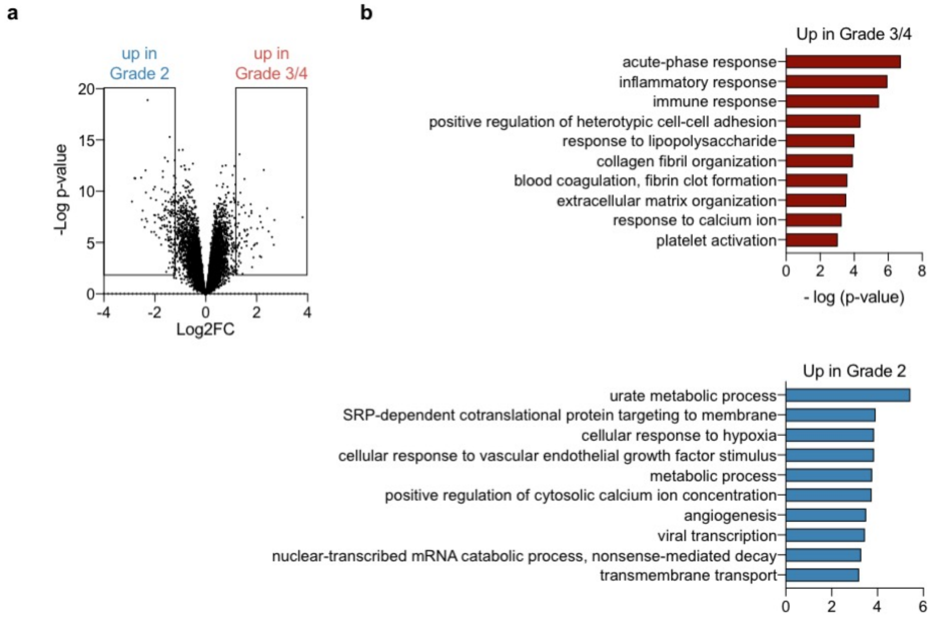

Supplementary Fig. 8: a, Differential gene expression in high/low grade tumor thrombus. Two-sided unpaired student t-test. **b**, enriched gene sets in high grade (grade 3/4) and low grade (grade 2) TT. Nominal p-value from GSEA analysis.

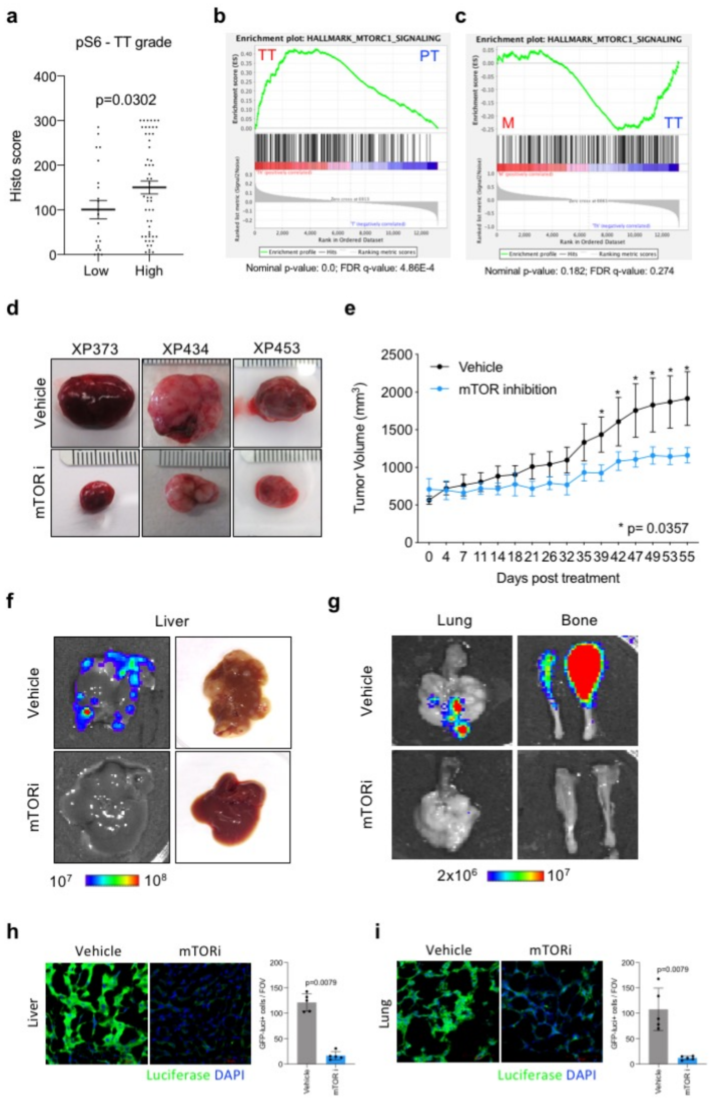

Supplementary Fig. 9: mTOR inhibition attenuates tumor growth and metastasis. **a,** Immunohistochemical quantification of phospho-S6 in low-grade and high-grade TT (n=65 patients, 81 samples). Data are presented as scatter dot plots and lines are at mean +/- SEM. Two-tailed Mann-Whitney *U*-test. **b-c**, Gene set enrichment analysis of mTOR signaling between PT and TT (**b**) and TT and M (**c**). **d,** Gross images of PDXs from TT (XP373 and XP453) and metastasis (XP434) treated with vehicle or mTOR inhibitor. **e,** Tumor growth curve of metastatic PDX line XP434 with vehicle or mTOR inhibitor (vehicle (n=5) and mTOR inhibitor (n=3) mice). Data are presented as mean +/- SEM. One-tailed Mann-Whitney *U*-test. **f,g,** Representative images highlighting reduced metastasis in liver (**f**), lung and bone (**g**) from mice treated with everolimus compared to vehicle. **h,i**, Representative luciferase immunofluorescence images and quantification of liver (**h**) and lung (**i**) sections (n=5 for each group). Data are presented as mean +/- SD. Two-tailed Mann-Whitney *U*-test.

Supplementary Table 1 Patient and pathological characteristics.

	N (%)
Median age at diagnosis (IQR)	62 (52-69)
Sex	
Female	30 (36.1%)
Male	53 (63.9%)
Ethnicity	
Hispanic	27 (32.9%)
Non-Hispanic	55 (67.1%)
Race	
Asian	1 (1.2%)
Black	7 (8.5%)
Native Hawaiian/Pacific Islander	1 (1.2%)
White	73 (89.0%)
Median BMI (IQR)	29.0 (25.0-33.1)
Median tumor size, cm (IQR)	8.9 (7.0-11.5)
Focality	
Multifocal	8 (9.9%)
Unifocal	73 (90.1%)
Histology	
RCC	
Chromophobe RCC	1 (1.2%)
Papillary RCC	1 (1.2%)
Unclassified RCC	7 (8.4%)
Clear cell RCC	73 (88.0%)
Non-RCC	
Leiomyosarcoma	1 (1.2%)
Sarcomatoid dedifferentiation	
Present	13 (15.9%)
Not identified	69 (84.1%)
Necrosis	
Present	58 (70.7%)
Not identified	24 (29.3%)
Tumor grade	
1	0 (0.0%)
2	4 (4.9%)
3	42 (51.9%)
4	35 (43.2%)
Margin involvement*	
Margins involved	25 (30.5%)
Margins uninvolved	57 (69.5%)
pT	
3a	37 (45.1%)
3b	28 (34.1%)
3c	9 (11.0%)

4	8 (9.8%)
pN	
pN1	15 (18.3%)
pN0 (or pNx)	67 (81.7%)
Metastases development	
At diagnosis	23 (27.7%)
After diagnosis	18 (21.7%)
Not observed	42 (51.6%)

*Malignant cells microscopically observed at distant surgical vein margin.

Supplementary Table 2 Architectural features in ccRCC, and their frequencies in primary tumor and in thrombus.

	**Frequency (%)**	
	**Tumor (n = 73)**	**Thrombus (n= 71)**
**Architectural Features**		
Microcystic	8 (11.0%)	0 (0%)
Tubular/Acinar	13 (17.8%)	4 (5.6%)
Compact small nests	36 (49.3%)	13 (18.3%)
Bleeding follicles	4 (5.5%)	0 (0%)
Large nests	37 (50.7%)	17 (23.9%)
Alveolar	38 (52.1%)	21 (29.6%)
Papillary/Pseudopapillary	14 (19.2%)	6 (8.5%)
Thick trabecular/Insular	44 (60.3%)	11 (15.5%)
Solid sheet	58 (79.5%)	33 (46.5%)
Median patterns present (IQR)	3 (2-5)	1 (1-2)

Supplementary Table 3 Clinicopathological characteristics of ccRCC cohort. Association between features and metastasis evaluated with a Fisher's exact test (categorical features) and Student's t-test (continuous measures).

	TT^{NM} **(n = 38)**	TT^{M} **(n = 35)**	***p***
Median age at diagnosis, years (IQR)	64.5 (55-69)	59 (52-66)	0.096
Sex			
Female	13 (34.2%)	11 (31.4%)	0.80
Male	25 (65.8%)	24 (68.6%)	
Ethnicity			
Hispanic	11 (29.7%)	15 (42.9%)	0.25
Non-Hispanic	26 (70.3%)	20 (57.1%)	
Race			
Asian	1 (2.7%)	0 (0.0%)	0.62
Black	2 (5.4%)	2 (5.7%)	
White	34 (91.9%)	33 (94.3%)	
Median tumor size, cm (IQR)	7.9 (6.7-10.2)	9.5 (7.5-13.0)	0.086
Focality			
Unifocal	34 (91.9%)	31 (88.6%)	0.63
Multifocal	3 (8.1%)	4 (11.4%)	
Margin involvement	11 (28.9%)	11 (31.4%)	0.82
pT			
3a	25 (65.8%)	8 (22.9%)	**0.0016**
3b	10 (26.3%)	15 (42.9%)	
3c	2 (5.3%)	6 (17.1%)	
4	1 (2.6%)	6 (17.1%)	
pN			
pN0/pNx	37 (97.4%)	25 (71.4%)	**0.0020**
pN1	1 (2.6%)	10 (28.6%)	
Sarcomatoid dedifferentiation	1 (2.6%)	9 (25.7%)	**0.0042**
Tumor necrosis present	20 (52.6%)	31 (88.6%)	**0.0008**
Tumor grade			
2	4 (10.5%)	0 (0%)	**<0.0001**
3	27 (71.1%)	11 (31.4%)	
4	7 (18.4%)	24 (68.6%)	
Thrombus grade			
2	21 (55.3%)	4 (12.1%)	**0.0007**
3	13 (34.2%)	23 (69.7%)	
4	4 (10.5%)	6 (18.2%)	
Indolent pattern present in PT	29 (76.3%)	21 (60.0%)	0.13
Indolent pattern present in TT	13 (34.2%)	3 (9.1%)	**0.012**
Aggressive pattern present in PT	33 (86.8%)	35 (100%)	**0.026**
Renal cortical parenchyma infiltration	13 (34.2%)	29 (82.9%)	**<0.0001**

Supplementary Table 4 Multivariate Cox proportional hazards model for time to metastasis diagnosis. Final variables chosen using backwards selection of the factors that met 0.20 significance cutoff on univariate analysis.

	**Events / Total**	**Hazard Ratio (95% CI)**	**Cox *p***
pT			
3a	8/33	Reference	0.045
3b	15/25	2.94 (1.15, 7.50)	
3c	6/8	5.07 (1.57, 16.42)	
4	4/5	1.62 (0.41, 6.41)	
pN			
pN0/pNx	23/60	Reference	0.0085
pN1	10/11	3.26 (1.35, 7.86)	
Sarcomatoid			
Not identified	25/62	Reference	0.0027
Present	8/9	5.27 (1.78, 15.58)	
Thrombus grade			
Low grade (G2)	4/25	Reference	0.0033
High grade (G3-G4)	29/46	6.42 (1.86, 22.20)	

Supplementary Table. 5: List of primers

Gene	Direction	Sequence
FOS	Forward	CCGGGGATAGCCTCTCTTACT
	Reverse	CCAGGTCCGTGCAGAAGTC
JUNB	Forward	ACGACTCATACACAGCTACGG
	Reverse	GCTCGGTTTCAGGAGTTTGTAGT
EGR1	Forward	ACCCCTCTGTCTACTATTAAGGC
	Reverse	TGGGACTGGTAGCTGGTATTG
ATF3	Forward	CCTCTGCGCTGGAATCAGTC
	Reverse	TTCTTTCTCGTCGCCTCTTTTT

Supplementary Table. 6: List of 332 driver genes

ABI1	CACNA1D	DDX6	FIP1L1	KIF5B	MYO5A	POLQ	SIRPA	VAV1
ABL1	CALR	DGCR8	FLCN	KLF4	N4BP2	PPM1D	SIX2	VHL
ABL2	CAMTA1	DICER1	FNBP1	KLF6	NBN	PPP2R1A	SMARCA4	WIF1
ACSL3	CANT1	DNAJB1	FOXO3	KMT2A	NCOA2	PRCC	SMC1A	WNK2
ACVR2A	CARD11	DNM2	FUBP1	KMT2C	NCOR1	PRDM16	SMO	WRN
AFF1	CASP3	DNMT3A	FUS	KMT2D	NCOR2	PRDM2	SPEN	WT1
AFF3	CBLB	EGFR	GAS7	KNSTRN	NDRG1	PREX2	SRGAP3	ZEB1
AFF4	CBLC	EIF3E	GLI1	LASP1	NF2	PRRX1	SRSF2	ZFHX3
AKAP9	CD74	ELF3	GMPS	LATS2	NFE2L2	PSIP1	SS18	ZMYM3
AKT2	CDC73	ELL	GNA11	LCP1	NFKBIE	PTEN	STAG2	ZNF384
ALK	CDH1	EML4	GOPC	LEF1	NIN	PTPN6	STAT3	ZNF521
AMER1	CDH10	EP300	GPC3	LHFP	NKX2-1	PTPRB	STIL	ZNRF3
APC	CDH11	EPAS1	GPHN	LIFR	NOTCH1	PTPRC	STK11	
AR	CDK12	ERBB3	GRIN2A	LMNA	NOTCH2	PTPRD	SUZ12	
ARHGAP26	CEP89	ERBB4	H3F3B	LPP	NRG1	PTPRT	TAL1	
ARHGEF10L	CHD2	ERC1	HEY1	LRIG3	NSD1	RAC1	TCF12	
ARID1A	CHD4	ERCC3	HIF1A	LRP1B	NTRK1	RAD51B	TCF7L2	
ARID1B	CHST11	ERCC4	HIP1	LZTR1	NUMA1	RAF1	TEC	
ARID2	CIC	ERCC5	HIST1H3B	MAML2	NUP214	RANBP2	TET1	
ARNT	CLIP1	ESR1	HNF1A	MAP3K1	NUP98	RAP1GDS1	TET2	
ASPSCR1	CLP1	ETNK1	HOXD13	MAP3K13	NUTM1	RBM10	TFEB	
ASXL1	CLTC	ETV4	HSP90AA1	MAPK1	NUTM2B	RBM15	TFRC	
ASXL2	CLTCL1	EXT2	HSP90AB1	MDM2	P2RY8	REL	TGFBR2	
ATP2B3	CNBP	EZR	IGF2BP2	MDS2	PABPC1	RET	THRAP3	
ATR	CNTNAP2	FAM131B	IKBKB	MED12	PAFAH1B2	RFWD3	TMPRSS2	
ATRX	COL1A1	FANCA	IL7R	MITF	PAX3	RHOA	TNC	
AXIN2	COL3A1	FANCD2	IRF4	MKL1	PAX5	RNF213	TNFAIP3	
BAP1	CREB1	FANCG	ITK	MLH1	PAX7	RNF43	TOP1	
BAZ1A	CREBBP	FAS	JAK2	MLLT1	PAX8	RPL5	TP53	
BCL11B	CRTC1	FAT1	JAK3	MLLT10	PBRM1	RUNX1T1	TPM4	
BCL6	CSMD3	FAT4	JUN	MLLT3	PCM1	SALL4	TPR	
BCOR	CTCF	FBLN2	KAT6A	MN1	PDE4DIP	SBDS	TRAF7	
BCORL1	CTNNA2	FBXO11	KAT6B	MPL	PDGFRA	SDHA	TRIM24	
BIRC3	CTNNB1	FBXW7	KCNJ5	MSH2	PDGFRB	SDHC	TRIM33	
BIRC6	CUL3	FES	KDM5A	MSH6	PER1	SETD2	TRRAP	
BLM	CUX1	FGFR1	KDM5C	MTOR	PIK3CA	SETDB1	TSC1	
BMPR1A	CYLD	FGFR1OP	KDM6A	MUC16	PIK3CB	SF3B1	TSC2	
BRCA1	DCTN1	FGFR2	KDSR	MUC4	PML	SFPQ	UBR5	
BRCA2	DDIT3	FGFR4	KEAP1	MUTYH	PMS2	SGK1	USP6	
BRIP1	DDR2	FH	KIAA1549	MYCN	POLE	SH3GL1	USP8	
