# Supplementary material for: Determinants of renal cell carcinoma invasion and metastatic competence
Source: Nat Commun. 2021 Oct 4;12:5760. doi: 10.1038/s41467-021-25918-4 (PMC8490399; doi:10.1038/s41467-021-25918-4)
Supplement: Supplementary file 2 — Description of Additional Supplementary Files [file 41467_2021_25918_MOESM2_ESM.pdf]

**Description of Additional Supplementary Files**

**File Name: Supplementary Data 1**
**Description:** Complete list of enriched pathways in TT. nominal p-value (NOM p-val), False discovery rate q-value (FDR q-val), and Familywise-error rate p-value (FWER p-val) from GSEA analysis.
