# Supplementary material for: Determinants of renal cell carcinoma invasion and metastatic competence
Source: Nat Commun. 2021 Oct 4;12:5760. doi: 10.1038/s41467-021-25918-4 (PMC8490399; doi:10.1038/s41467-021-25918-4)
Supplement: Supplementary file 4 — Reporting Summary [file 41467_2021_25918_MOESM4_ESM.pdf]

nature portfolio

Corresponding author(s): Srinivas Malladi

Last updated by author(s): Jul 20, 2021

Reporting Summary

Nature Portfolio wishes to improve the reproducibility of the work that we publish. This form provides structure for consistency and transparency in reporting. For further information on Nature Portfolio policies, see our Editorial Policies and the Editorial Policy Checklist.

Statistics

For all statistical analyses, confirm that the following items are present in the figure legend, table legend, main text, or Methods section.

n/a	Confirmed	
☐	☒	The exact sample size (*n*) for each experimental group/condition, given as a discrete number and unit of measurement
☐	☒	A statement on whether measurements were taken from distinct samples or whether the same sample was measured repeatedly
☐	☒	The statistical test(s) used AND whether they are one- or two-sided<br>*Only common tests should be described solely by name; describe more complex techniques in the Methods section.*
☒	☐	A description of all covariates tested
☒	☐	A description of any assumptions or corrections, such as tests of normality and adjustment for multiple comparisons
☐	☒	A full description of the statistical parameters including central tendency (e.g. means) or other basic estimates (e.g. regression coefficient) AND variation (e.g. standard deviation) or associated estimates of uncertainty (e.g. confidence intervals)
☐	☒	For null hypothesis testing, the test statistic (e.g. *F*, *t*, *r*) with confidence intervals, effect sizes, degrees of freedom and *P* value noted<br>*Give P values as exact values whenever suitable.*
☒	☐	For Bayesian analysis, information on the choice of priors and Markov chain Monte Carlo settings
☒	☐	For hierarchical and complex designs, identification of the appropriate level for tests and full reporting of outcomes
☒	☐	Estimates of effect sizes (e.g. Cohen's *d*, Pearson's *r*), indicating how they were calculated

Our web collection on statistics for biologists contains articles on many of the points above.

Software and code

Policy information about availability of computer code

Data collection

For mutation calling, we used the QBRC mutation calling pipeline (https://github.com/Somatic-pipeline/Somatic-pipeline). Exome-seq reads were aligned to the human reference genome by BWA-MEM (version>=0.7.15). Picard (2.20.0)was used to add read group information and Sambamba (0.8.0) was used to mark PCR duplicates. The GATK toolkit (4.0) was used to perform base quality score recalibration and local realignment around Indels. MuTect (1.1.6), VarScan (2.4.2), Shimmer (0.2), SpeedSeq (0.1.2), Manta (version>=1.4.0), Strelka (version>=2.8.3), and Lofreq (version >=2.1.3) were used to call SNPs and Indels. A mutation that was repeatedly called by any three of these softwares was retained. RNA-seq reads were aligned to the human reference genome GRCh38 (hg38) using STAR with the parameters "--runThreadN 48 --outSAMtype BAM Unsorted --outReandsUnmapped Fastx." featureCounts19 with parameters "--primary -O -t exon -g transcript_id -s 0 -T 48 --largestOverlap --minOverlap 3 --ignoreDup -p -P -B -C" was then used to measure gene expression levels. The human genome annotation file employed by featureCounts (1.6.0) was downloaded from the University of California Santa Cruz (UCSC) table browser under the RefSeq Gene track. We visualized and scanned immunoblot using Chemidoc Imaging System (Biorad). Cellular migration was quantified using IncuCyte software. Quantitative RT-PCR was performed using CFX Connect Real-Time PCR system (BioRad). For in vivo imaging data collection, we used IVIS Spectrum In Vivo Imaging System (Perkin Elmer) and Living Imaging sofware (version 4.4).

Data analysis

We carried out somatic copy number variation (CNV) analyses using CNVkit (0.9.4) with default parameters on paired tumor-normal sequencing data. CNVkit uses both on- and off-target sequencing reads to calculate log2 copy ratios across the genome for each sample and improves accuracy in copy number calling by applying a series of corrections Arm gain or loss was called when >50% of the chromosome exhibited copy number gain or loss. In order to have a chromosome arm level estimate of copy number (ranging from 0 to 4, whereas the normal would be 2), we calculated the weighted average of copy numbers from each gene within the target chromosome arm. The weights of each gene were derived from the 'Bin-level Log2 ratios' outputted by the CNVkit [https://github.com/etal/cnvkit], and the gene level copy numbers were calculated using the QBRC Somatic Pipeline. Loss status was called if the chromosomal arm level copy number is <= 1 and a gain status will be called if the chromosomal arm level copy number is >=3. Read per kilobase million (RPKM) values were calculated from gene read counts. RPKM values were then log2-transformed. We analyzed Next Generation sequencing data using R software (version 3.6.0) and Morpheus (Broad Institute). We analyzed and quantified in vivo bioimaging data using Living image software. We conduct statistical analyses using Prism software v8.0 (GraphPad). Ranked Gene Set Enrichment Analysis (GSEA) was performed with software version 4.0.3.

Default setting was used except for “Collapse/Remap dataset to gene symbols” set to “No Collapse” and “Phenotype” permutation type to “Gene_set”. Defined lists from broad institute were used: h.all.v.7.0.symbol.gmt [Hallmarks], c2.all.v.7.0.symbol.gmt [Curated], c3.all.v.7.0.symbol.gmt [motif], and c5.all.v.7.0.symbol.gmt [Gene Ontology]. Detailed statistical criteria have described in result and figure legends.

For manuscripts utilizing custom algorithms or software that are central to the research but not yet described in published literature, software must be made available to editors and reviewers. We strongly encourage code deposition in a community repository (e.g. GitHub). See the Nature Portfolio guidelines for submitting code & software for further information.

Data

Policy information about availability of data

All manuscripts must include a data availability statement. This statement should provide the following information, where applicable:

- Accession codes, unique identifiers, or web links for publicly available datasets
- A description of any restrictions on data availability
- For clinical datasets or third party data, please ensure that the statement adheres to our policy

The raw whole exome/RNA-seq data for this manuscript are available through European Genome-phenome Archive (EGA) under the accession number EGAS00001005511 and EGAS00001005512

Field-specific reporting

Please select the one below that is the best fit for your research. If you are not sure, read the appropriate sections before making your selection.

[x] Life sciences [ ] Behavioural & social sciences [ ] Ecological, evolutionary & environmental sciences

For a reference copy of the document with all sections, see nature.com/documents/nr-reporting-summary-flat.pdf

Life sciences study design

All studies must disclose on these points even when the disclosure is negative.

Sample size	In general, no calculations were done to determine sample size. Sample size was determined based on standards for cell line and animal studies from our previous published studies (Malladi et al., 2016).
Data exclusions	No data were excluded from the analysis
Replication	At least two biological independents with similar results were performed.
Randomization	Experiments were all randomized.
Blinding	In general, the investigators were blind at the time of experiment execution and data acquisition. For in vivo experiments, mice were randomized to everolimus treatment groups after tumor injection and data were collected under blinded experimental conditions.

Reporting for specific materials, systems and methods

We require information from authors about some types of materials, experimental systems and methods used in many studies. Here, indicate whether each material, system or method listed is relevant to your study. If you are not sure if a list item applies to your research, read the appropriate section before selecting a response.

Materials & experimental systems

n/a	Involved in the study	
[ ]	[x]	Antibodies
[ ]	[x]	Eukaryotic cell lines
[x]	[ ]	Palaeontology and archaeology
[ ]	[x]	Animals and other organisms
[ ]	[x]	Human research participants
[x]	[ ]	Clinical data
[x]	[ ]	Dual use research of concern

Methods

n/a	Involved in the study	
[x]	[ ]	ChIP-seq
[x]	[ ]	Flow cytometry
[x]	[ ]	MRI-based neuroimaging

Antibodies

Antibodies used	CD31 Antibody Agilent Technologies, Inc Cat# M082301-2<br>ERG Antibody Biocare Medical Cat# CM 421 A, C<br>CD163 Antibody Biocare Medical Cat# CM 353 AK, CK

	Phospho-S6 Ribosomal Protein (Ser235/236) Antibody Cell Signaling Technology Cat# 2211<br>Phospho-CREB (Ser133) Antibody Cell Signaling Technology Cat# 9198<br>Beta Actin Antibody Abcam Cat# ab49900<br>Goat anti-Rabbit IgG, (H+L) HRP conjugate antibody Millipore Cat# AP307P
Validation	CD31 Antibody (Monoclonal Mouse Anti-Human CD31, Endothelial Cell, Clone JC70A,) is intended for use in immunohistochemistry (IHC). The antibody primarily labels endothelial cells. Results aid in the classification of malignant vascular disorders, including angiosarcomas. Differential classification is aided by the results from a panel of antibodies. The clinical interpretation of any staining or its absence should be complemented by morphological studies using proper controls and should be evaluated within the context of the patient's clinical history and other diagnostic tests by a qualified pathologist. This antibody is intended to be used after the primary diagnosis of tumor has been made by conventional histopathology using nonimmunologic histochemical stains.<br><br>ERG Antibody is a mouse monoclonal antibody that is intended for laboratory use in the qualitative identification of ERG protein by immunohistochemistry (IHC) in formalin-fixed paraffin-embedded (FFPE) human tissues. The clinical interpretation of any staining or its absence should be complemented by morphological studies using proper controls and should be evaluated within the context of the patient's clinical history and other diagnostic tests by a qualified pathologist. It is react with human and others are not tested.<br><br>CD163 Antibody is a mouse monoclonal antibody that is intended for laboratory use in the qualitative identification of CD163 protein by immunohistochemistry (IHC) in formalin-fixed paraffin-embedded (FFPE) human tissues. The clinical interpretation of any staining or its absence should be complemented by morphological studies using proper controls and should be evaluated within the context of the patient's clinical history and other diagnostic tests by a qualified pathologist. It is react with human and others are not tested<br><br>Phospho-S6 Ribosomal Protein (Ser235/236) Antibody detects endogenous levels of ribosomal protein S6 only when phosphorylated at serine 235 and 236. This antibody does not detect ribosomal protein S6 phosphorylated at other sites. It is react with human, mouse, monkey, rat, S. cerevisiae. Application: Western blot, Immunoprecipitation, Immunohistochemistry, Immunofluorescence, and Flow cytometry.<br><br>Phospho-CREB (Ser133) (87G3) Rabbit monoclonal antibody detects endogenous levels of CREB only when phosphorylated at serine 133. The antibody also detects the phosphorylated form of the CREB-related protein, ATF-1. It is react with human, mouse, and rat. Application: Western blot, Immunohistochemistry, Immunofluorescence, Flow cytometry, and Chromatin Immunoprecipitation.<br><br>Beta Actin Antibody is mouse monoclonal antibody conjugated with HRP. It is react with mouse, rat, cow, dog, human, and african green monkey. Application: Western blot. Synthetic peptide corresponding to beta Actin (N terminal) conjugated to keyhole limpet haemocyanin. Sequence: DDDIAALVIDNGSGK<br><br>Goat anti-Rabbit IgG, (H+L) HRP conjugate antibody is an affinity purified antibody from goat. The purified antibody is conjugated to horseradish peroxidase (HRP) and stabilized in buffer. It is react with rabbit. Application: WB, ELISA, IHC, and IC.

Eukaryotic cell lines

Policy information about cell lines

Cell line source(s)	769-P and 786-O (ATCC). A498, Caki-1 and UMRC-2 cells (ATCC, provided by Qing Zhang, UT Southwestern)
Authentication	As all cell lines were originally purchased from certified commercial vendors, no further authentication was performed.
Mycoplasma contamination	All lines were tested negative to be negative for mycoplasma (Universal Mycoplasma Detection kit, ATCC)
Commonly misidentified lines (See ICLAC register)	No commonly misidentified cell lines were used in the study.

Animals and other organisms

Policy information about studies involving animals; ARRIVE guidelines recommended for reporting animal research

Laboratory animals	NOD.CB17-Prkdcscid Il2rgtm1Wjl/SzJ (NSG) female mice from 4-6 weeks of age. All mice were housed in barrier facilities maintained in individually ventilated microisolator cages. All caging equipment was autoclaved and all feed was commercial irradiated diet. Cage manipulations and animal handling was performed in cage change stations or biosafety cabinets. Automated watering systems provided water that was purified through reverse osmosis and chlorination. The standard white light cycle was from 6:00AM to 5:59PM and dark cycle was from 6:00PM to 5:59AM. 23–25 °C temperature and 50–60% relative humidity were maintained.
Wild animals	The study did not involve wild animals.
Field-collected samples	The study did not involve samples collected from the fields.
Ethics oversight	All procedures were performed in strict accordance with the Guide for Care and Use of Laboratory Animals approved by the UT Southwestern Institutional Animal Care and Use Committee (IACUC).

Note that full information on the approval of the study protocol must also be provided in the manuscript.

Human research participants

Policy information about studies involving human research participants

Population characteristics	Patients that enrolled in the study provided written consent allowing the use of discarded surgical samples for research purposes and genetic studies according to an Institutional Review Board-approved protocol. Surgical schedules were screened weekly for RCC patients undergoing tumor excisions or biopsies. Inclusion criteria were largely based off imaging; evidence of local invasion, metachronous primaries, recurrent tumors, lymphadenopathy, or distant metastasis. Population characteristics are like this: Female (36.1%) and male (63.9%). Median age at diagnosis is 62 (range 52-69). Hispanic (32.9%) and non-Hispanic (67.1%). Asian (1.2%), Black (8.5%), Native Hawaiian/Pacific Islander (1.2%), and White (89.0%). Detailed characteristics are described in Supplementary Table. 1.
Recruitment	Patients were included in the study based on tumor sample availability, especially for tumor thrombus growth. The study was a randomized double blind study where both participants, clinical investigators and site personnel as well as sponsor teams were blinded to preclude opportunity for self selection bias. Thus any potential selection bias in this cohort is expected to have been minimal, and of no significant impact on the results of this study.
Ethics oversight	Ethics approval was obtained from The University of Texas Southwestern Medical Center Tissue Resource [STU 102010-051], Kidney Cancer New Pathway Discovery Project [STU 012011-190], and Comprehensive Database for Patients with Kidney Tumor [STU 022015-015].

Note that full information on the approval of the study protocol must also be provided in the manuscript.
