# Supplementary material for: Determinants of renal cell carcinoma invasion and metastatic competence
Source: Nat Commun. 2021 Oct 4;12:5760. doi: 10.1038/s41467-021-25918-4 (PMC8490399; doi:10.1038/s41467-021-25918-4)
Supplement: Supplementary file 5 — Source Data [file 41467_2021_25918_MOESM5_ESM.zip › 309048_2_data_set_5808677_qxn4sf.pptx]

## Slide 1
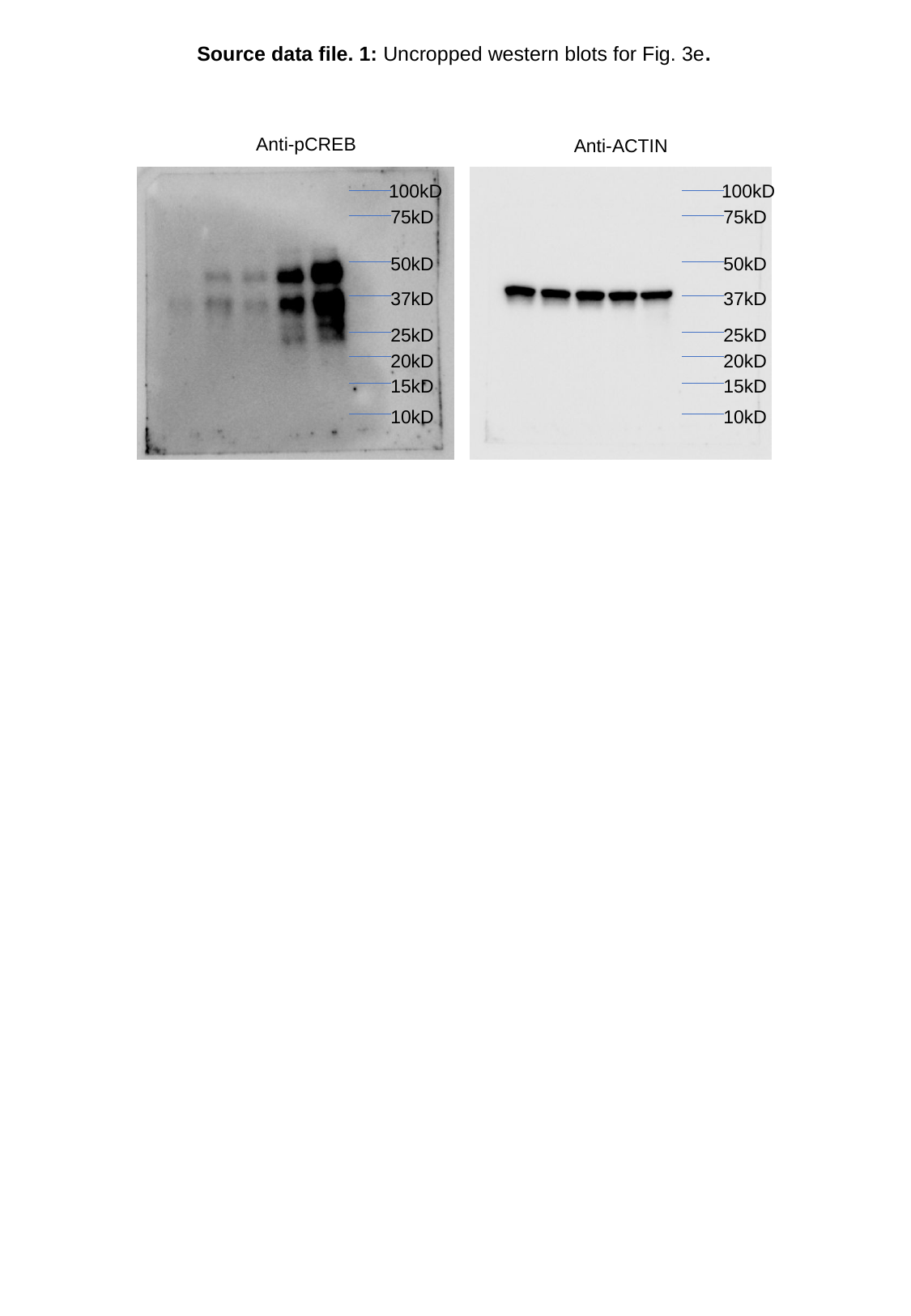

Source data file. 1: Uncropped western blots for Fig. 3e.
Anti-pCREB
Anti-ACTIN
100kD
75kD
50kD
37kD
25kD
20kD
15kD
10kD
100kD
75kD
50kD
37kD
25kD
20kD
15kD
10kD
